# Sex Differences in the Relationship of Socioeconomic Position With Cardiovascular Disease, Cardiovascular Risk Factors, and Estimated Cardiovascular Disease Risk: Results of the German National Cohort

**DOI:** 10.1161/JAHA.124.038708

**Published:** 2025-02-25

**Authors:** Ilais Moreno Velásquez, Sanne A. E. Peters, Nico Dragano, Karin Halina Greiser, Marcus Dörr, Beate Fischer, Klaus Berger, Anke Hannemann, Renate B. Schnabel, Matthias Nauck, Susanne Göttlicher, Susanne Rospleszcz, Stefan N. Willich, Lilian Krist, Matthias B. Schulze, Kathrin Günther, Tilman Brand, Tamara Schikowski, Carina Emmel, Börge Schmidt, Karin B. Michels, Rafael Mikolajczyk, Alexander Kluttig, Volker Harth, Nadia Obi, Stefanie Castell, Carolina J. Klett‐Tammen, Wolfgang Lieb, Heiko Becher, Volker Winkler, Heike Minnerup, André Karch, Claudia Meinke‐Franze, Michael Leitzmann, Michael J. Stein, Barbara Bohn, Ben Schöttker, Kira Trares, Annette Peters, Tobias Pischon

**Affiliations:** ^1^ Max‐Delbrück‐Center for Molecular Medicine in the Helmholtz Association (MDC) Molecular Epidemiology Research Group Berlin Germany; ^2^ The George Institute for Global Health, School of Public Health Imperial College London UK; ^3^ Julius Centre for Health Sciences and Primary Care University Medical Centre Utrecht the Netherlands; ^4^ Institute of Medical Sociology, Centre for Health and Society, Medical Faculty and University Hospital Heinrich Heine University Düsseldorf Germany; ^5^ German Cancer Research Center in the Helmholtz Association DKFZ Heidelberg Germany; ^6^ Department of Internal Medicine University Medicine Greifswald Germany; ^7^ German Center of Cardiovascular Research (DZHK) Partner Site Greifswald Germany; ^8^ Department of Epidemiology and Preventive Medicine University of Regensburg Germany; ^9^ Institute of Epidemiology and Social Medicine University of Münster Germany; ^10^ Institute of Clinical Chemistry and Laboratory Medicine University Medicine Greifswald Germany; ^11^ Department of Cardiology, University Heart & Vascular Center Hamburg University Medical Center Hamburg‐Eppendorf Hamburg Germany; ^12^ German Centre for Cardiovascular Research (DZHK) Partner Site Hamburg/Kiel/Luebeck Hamburg Germany; ^13^ Institute of Epidemiology, Helmholtz Zentrum München ‐ German Research Center for Environmental Health Neuherberg Germany; ^14^ Department of Diagnostic and Interventional Radiology, University Medical Center Freiburg, Faculty of Medicine University of Freiburg Germany; ^15^ Institute of Social Medicine, Epidemiology and Health Economics Charité ‐ Universitätsmedizin Berlin Germany; ^16^ Department of Molecular Epidemiology German Institute of Human Nutrition Potsdam Rehbruecke Nuthetal Germany; ^17^ Institute of Nutritional Science University of Potsdam Nuthetal Germany; ^18^ Leibniz Institute for Prevention Research and Epidemiology‐BIPS Bremen Germany; ^19^ Department of Epidemiology IUF‐Leibniz Research Institute for Environmental Medicine Düsseldorf Germany; ^20^ Institute for Medical Informatics, Biometry and Epidemiology Essen University Hospital Essen Germany; ^21^ Institute for Prevention and Cancer Epidemiology, Faculty of Medicine and Medical Center University of Freiburg Germany; ^22^ Institute for Medical Epidemiology, Biometrics, and Informatics, Interdisciplinary Center for Health Sciences Medical Faculty of the Martin‐Luther University Halle‐Wittenberg Halle Germany; ^23^ Institute for Occupational and Maritime Medicine Hamburg (ZfAM) University Medical Centre Hamburg‐Eppendorf (UKE) Hamburg Germany; ^24^ Department for Epidemiology Helmholtz Centre for Infection Research Braunschweig Germany; ^25^ Institute of Epidemiology University of Kiel Germany; ^26^ Institute of Global Health University Hospital Heidelberg Germany; ^27^ Institute for Community Medicine University Medicine Greifswald Germany; ^28^ NAKO e.V. Heidelberg Germany; ^29^ Division of Clinical Epidemiology and Aging Research German Cancer Research Center Heidelberg Germany; ^30^ Chair of Epidemiology, Institute for Medical Information Processing, Biometry and Epidemiology, Medical Faculty Ludwig‐Maximilians‐Universität München Munich Germany; ^31^ German Center for Cardiovascular Research (DZHK), Partner Site Munich Germany; ^32^ Max‐Delbrück‐Center for Molecular Medicine in the Helmholtz Association (MDC) Biobank Technology Platform Berlin Germany; ^33^ Berlin Institute of Health (BIH) at Charité‐Universitätsmedizin Berlin Core Facility Biobank Berlin Germany; ^34^ Charité‐Universitätsmedizin Berlin, Corporate Member of Freie Universität Berlin and Humboldt‐Universität zu Berlin Germany; ^35^ German Center for Cardiovascular Research (DZHK), Partner Site Berlin Germany

**Keywords:** cardiovascular disease, cardiovascular risk, educational attainment, income, socioeconomic position, Cardiovascular Disease, Epidemiology, Primary Prevention, Women

## Abstract

**Background:**

Using data from the largest German cohort study, we aimed to investigate sex differences in the relationship of socioeconomic position (SEP) with cardiovascular disease (CVD), CVD risk factors, and estimated CVD risk.

**Methods and Results:**

A total of 204 780 (50.5% women) participants from the baseline examination of the population‐based NAKO (German National Cohort) were included. Logistic, multinomial, and linear regression models were used to estimate sex‐specific odds ratios (ORs) and β coefficients with 95% CIs of CVD, CVD risk factors, and very high‐risk score (Systemic Coronary Risk Estimation‐2) for CVD associated with SEP. Women‐to‐men ratios of ORs (RORs) with 95% CIs were estimated. In women compared with men, low versus high SEP (educational attainment and relative income) was more strongly associated with myocardial infarction, hypertension, obesity, overweight, elevated blood pressure, antihypertensive medication, and current alcohol consumption, but less strongly with current and former smoking. In women with the lowest versus highest educational level, the OR for a very high 10‐year CVD risk was 3.61 (95% CI, 2.88–4.53) compared with 1.72 (95% CI, 1.51–1.96) in men. The women‐to‐men ROR was 2.33 (95% CI, 1.78–3.05). For the comparison of low versus high relative income, the odds of having a very high 10‐year CVD risk was 2.55 (95% CI, 2.04–3.18) in women and 2.25 (95% CI, 2.08–2.42) in men (women‐to‐men ROR, 1.31 [95% CI, 1.05–1.63]).

**Conclusions:**

In women and men, there was an inverse relationship between indicators of SEP and the likelihood of having several CVD risk factors and a very high 10‐year CVD risk. This association was stronger in women, suggesting that CVD risk is more strongly influenced by SEP in women compared with men.

**Clinical Perspective**

**What Is New?**

- To our knowledge, no previous study has comprehensively investigated sex differences in cardiovascular disease across the socioeconomic gradient in a contemporary German population.
- In women, we observed stronger inverse associations between socioeconomic position and the likelihood of having several cardiovascular disease risk factors and a very high 10‐year cardiovascular disease risk, suggesting that cardiovascular disease risk is more strongly influenced by socioeconomic position in women compared with men.

**What Are the Clinical Implications?**

- Our results support the need for tailored sex‐specific risk assessment strategies and interventions to reduce socioeconomic inequalities in estimated cardiovascular risk.

**Nonstandard Abbreviations and Acronyms**
NAKO: German National Cohort
PCE: pooled cohort equation
ROR: ratio of odds ratios
SBP: systolic blood pressure
SCORE2: Systemic Coronary Risk Estimation‐2
SEP: socioeconomic position

A growing body of studies reported meaningful sex differences across the spectrum of cardiovascular disease (CVD) and cardiovascular risk factors, prompting the need for sex‐specific guidelines for CVD prevention.^1^, ^2^ However, the biological, clinical, and socioeconomic differences of CVD between women and men are still under investigation.

Sex differences in CVD have been documented, from atherosclerotic plaque composition to clinical presentation of acute coronary syndromes, with additional symptoms in women.^3^, ^4^ Men <65 years of age have higher absolute atherosclerotic CVD event rates compared with women. Nevertheless, in most European countries, from 1990 to 2019, the relative age‐standardized CVD mortality rate decrease was slightly greater in men than women.^2^, ^5^ Moreover, cardiovascular risk factors may have a sex‐specific influence on CVD risk. Hypertension, smoking intensity, and type 2 diabetes have been more strongly associated with the risk of myocardial infarction (MI) in women than in men, and at an early age, women have steeper increases in blood pressure than men.^6^, ^7^

Educational deprivation has repeatedly been related to CVD risk^8^; however, the underlying pathways remain elusive and may affect sexes differently.^9^ For example, a meta‐analysis indicated a greater excess risk of fatal and nonfatal coronary heart disease (CHD) and CVD associated with lower educational attainment in women versus men.^10^ Yet, there was no evidence of sex differences in the excess risk of stroke, nor when considering other relevant indicators of social deprivation such as income.^10^ Although associations of low education and income with incident CVD have shown higher effect sizes in women than men compared with high education and income,^11^ there was no evidence of an excess risk in women. In European populations, inequalities in the distribution of risk factors accounted for over a third of the CHD educational class gradient in both women and men.^9^ Cigarette smoking was identified as a strong mediator of incident CHD inequalities among men, whereas high‐density lipoprotein (HDL) cholesterol was a key mediator among women.^9^

The latest guideline from the European Society of Cardiology for CVD risk estimation recommends the use of the recent Systemic Coronary Risk Estimation‐2 (SCORE2) algorithm, tailored to European populations.^12^ SCORE2 allows for estimating the sex‐specific 10‐year risk of CVD for people 40 to 69 years of age based on established risk factors including age, smoking, systolic blood pressure (SBP), total cholesterol, and HDL cholesterol.^13^ Yet, evidence on sex differences of socioeconomic position (SEP) in relation to CVD risk estimation using SCORE2 remains limited. In addition, little is known on whether sex differences and heterogeneity exist in the association of SEP across a spectrum of CVD and CVD risk factors in contemporary German populations.

Understanding the burden of CVD in women and men, and whether there are currently differences in risk factor control, is essential for informing policymakers and planning of health care delivery. Furthermore, determining whether sex differences in CVD occur across the socioeconomic gradient has public health implications, because socioeconomic circumstances and their effects can be influenced by policies at various levels.^14^ Using cross‐sectional data from the largest German cohort study, we aimed to investigate sex differences in the relationship of indicators of SEP with CVD, risk factors for CVD, and estimated CVD risk. We also examined to what extent differences in CVD risk factors across SEP and sex may account for differences in the relationships of SEP with CVD risk between sexes.

## METHODS

### Data Access and Responsibility

Access to and use of NAKO data and biosamples can be obtained via an electronic application portal (https://transfer.nako.de/transfer/index). The codes that support the findings of this study are available from the corresponding author upon reasonable request.

The German National Cohort [NAKO Gesundheitsstudie (NAKO)] is a large population‐based, prospective, and ongoing cohort study designed to investigate risk factors, mechanistic pathways, markers of early detection, and risk prediction for a broad range of diseases.^15^ A detailed description of the study design can be found elsewhere.^15^ Briefly, from 2014 to 2019, NAKO enrolled 205 415 adult participants 20 to 69 years of age. Participants were randomly selected from population registries of 18 urban and rural regions across Germany. The design intended to recruit for both women and men 10% of participants in each 10‐year group between 20 and 39 years of age and 26.7% in each 10‐year group between 40 and 69 years of age. The overall response was 17%. The baseline examination included standardized interviews, self‐administered questionnaires, in‐depth physical and medical examinations with clinical biomarker measurements, and biosample collection. The study has been approved by the relevant ethics committees, and written informed consent was obtained from all participants.

Up to the time of the present analysis, 631 participants withdrew their consent to be part of the study. Their data were deleted and therefore not part of the present analyses. Furthermore, 4 participants had missing information on sex. Information on sex assigned at birth was received from the population registries and documented by the study nurse during the examination. The sex variable received was binary (since the end of 2018, Germany legally allows the option of choosing diverse as a sex marker in civil status entries at the registry office). Thus, we received data from 204 780 study participants (103 324 women, 101 456 men) for the baseline assessment.

### SEP Definition

Educational attainment, assessed during the interview, was our primary indicator of SEP, combining information on formal school and vocational training. Education was categorized according to the International Standard Classification of Education‐1997 (ISCED‐97) as low (International Standard Classification of Education levels 1–2), medium (International Standard Classification of Education levels 3–4), or high (International Standard Classification of Education levels 5–6).^16^ A total of 4280 study participants were still enrolled in school or vocational training and were not included in the main analysis. The average monthly net household income was gathered during the standardized face‐to‐face interviews, using 24 income categories. Income relative to the median equivalent household income (relative income) was calculated based on the net equivalent household income that considers the size of household and the net household income.^17^ Relative income was categorized according to the European Union Statistics on Income and Living Conditions as <60% (at risk of poverty), 60% to 79%, 80% to 99%, 100% to 149%, and ≥150% (high‐income groups).^17^

### Definition of CVD and Cardiovascular Risk Factors

The categorization and measurements of prevalent diseases, smoking status, alcohol consumption, antihypertensive medication intake, employment status, migration status,^18^ ethnicity, SBP and diastolic blood pressure,^19^ waist circumference, body fat,^15^ body mass index (BMI), triglycerides, total cholesterol, low‐density lipoprotein cholesterol, HDL cholesterol, glycated hemoglobin, and high‐sensitivity C‐reactive protein are described in Table S1.

### Estimated CVD Risk

In participants without previous CVD or diabetes, we estimated the sex‐specific 10‐year risk of fatal CVD (ie, deaths due to CHD, heart failure, stroke, sudden death) and nonfatal MI and stroke according to the SCORE2 algorithm, which is intended for individuals 40 to 69 years of age.^13^ In this algorithm, Germany is considered a moderate‐risk region. The variables included in the algorithm were age (years), smoking (current versus other), SBP (millimeters of mercury), total cholesterol (millimoles per liter), and HDL cholesterol (millimoles per litre).^20^ A total of 143 019 participants were considered for the analyses of the SCORE2 (Figure S1). A very high 10‐year CVD risk was defined as a predicted 10‐year CVD risk ≥7.5% (40–49 years of age) or ≥10% (50–69 years of age). These cutoff values are based on recommendations for age‐specific treatment thresholds given in the European Society of Cardiology guidelines.^12^

In a supplementary analysis, we estimated the sex‐specific 10‐year risk of atherosclerotic cardiovascular disease (nonfatal MI or CHD death, fatal or nonfatal stroke) in the CVD‐free population using the pooled cohort equation (PCE).^21^ Similarly, we used the Reynolds Risk Score to estimate the sex‐specific 10‐year CVD risk (MI, ischemic stroke, coronary revascularization, and cardiovascular death).^22^, ^23^ The PCE is used in individuals 40 to 79 years of age, whereas the Reynolds Risk Score is tailored to those >45 years of age. After applying all exclusion criteria, 140 370 and 60 073 study participants were available for the PCE and the Reynolds Risk Score, respectively (Figures S2 and S3). A high CVD risk was defined as an estimated 10‐year CVD risk of ≥10%.^21^, ^22^, ^23^

### Statistical Analysis

Baseline characteristics of the 204 780 participants are given as percentages for categorical variables, mean±SD for approximately normally distributed continuous variables, and median with interquartile range for nonnormally distributed continuous variables. We calculated the percentage of missing data for our variables of interest (Table S2). Handling of missing data was addressed by performing 20 iterations of a multiple imputation using chained equations. For triglycerides, low‐density lipoprotein cholesterol, and high‐sensitivity C‐reactive protein, complete‐case analyses were used, because these biomarkers have not been measured in all study centers, and imputation was therefore considered inappropriate. We calculated the sex‐specific frequencies of low moderate risk, high risk, and very high 10‐year risk of CVD using the SCORE2 algorithm in 2 age groups (40–50 and 50–69 years of age),^20^ and reported estimates in the 2 age groups combined.

### Sex Differences in CVD, CVD Risk Factors, and Estimated CVD Risk

Logistic and multinomial logistic regression models were used to estimate sex‐specific odds ratios (ORs) with 95% CIs for the association between SEP (exposure variables) and dichotomous (self‐reported diseases, SBP/diastolic blood pressure ≥140/90 mm Hg, antihypertensive medication intake, family history of MI, and a very high 10‐year CVD risk) and categorical (smoking, alcohol intake, BMI categories) outcomes, respectively. Linear regression analysis was used to estimate β coefficients with 95% CIs for the association of SEP with blood pressure, anthropometric measurements, and blood biomarkers. Log transformation was required for BMI, glycated hemoglobin, triglycerides, and high‐sensitivity C‐reactive protein, and estimates were back‐transformed and interpreted as the ratio of the geometric mean in the outcome in the lowest categories of SEP over the geometric mean of the outcome in high SEP groups. Reference categories were high education or high relative income. All of the main models, except those examining 10‐year CVD risk as an outcome, were age adjusted. We further evaluated migration status as a potential confounding factor in the association between education and CVD and risk factors. Estimates were considered to be statistically significant if the false discovery rate corrected *P* value was ≤0.05. However, we only report the corresponding unadjusted 95% CI.

We estimated women‐to‐men ratios of ORs (RORs) with 95% CIs for CVD, CVD risk factors, and very high CVD risk depending on SEP, using formulas reported elsewhere.^24^ The interaction terms of SEP with sex were used to obtain the women‐to‐men RORs for each category of SEP. Briefly, the SE of the OR was calculated by first taking the natural logarithm of the OR and 95% CI by sex, then calculating the SE of the sex‐specific natural logarithm ORs by taking the mean of the SE of the natural logarithm of the upper and lower 95% CIs, and finally calculating the sum of the sex‐specific variances to derive the SE of the natural logarithm OR by taking the square root.

Our primary model for the association between SEP and a very high CVD risk included no covariates that were components of SCORE2 to avoid overadjustments. In sensitivity analyses, however, we aimed to evaluate to what extent the association of education and income with very high CVD risk was influenced by other sociodemographic characteristics. We stratified the models by migration status and employment. We further evaluated the inclusion of age, smoking, SBP, and antihypertensive treatment individually in the crude models to explore how the differences observed were related to variations in the distribution of these factors among SEP categories. The associations between SEP and a very high CVD risk were replicated using the PCE and the Reynolds Risk Score algorithms using the population criteria and cutoffs specified with a complete‐case approach.

SAS Enterprise version 8.4 and R version 4.3.1 were used for statistical analyses.

## RESULTS

The proportion of women was 50.5%, and the mean age at the baseline examination was 49±13 years in the overall population (Table 1). Participants enrolled in education or vocational training (50.5% women) had a median age of 23 years (interquartile range, 22–25 years of age; age range, 19–70 years of age) and were excluded from further analyses.

**Table 1:** Baseline Characteristics of the German National Cohort Participants Baseline Assessment*

Characteristic	Women	Men
N (%)	103 324 (50.46)	101 456 (49.54)
Age, y, mean±SD†	49.80±12.73	49.91±12.76
Educational attainment, %‡		
Enrolled in education/vocational training§	2.24	2.25
Low	3.37	2.10
Medium	45.39	36.90
High	49.01	58.75
Monthly net equivalent income, euro, median (IQR)‡	1833.33 (1375.00–2533.33)	2033.33 (1433.33–2833.33)
Income relative to the median of Germany, %‡		
<60%	15.85	13.88
60%–79%	16.04	12.78
80%–99%	15.37	13.26
100%–149%	30.68	31.24
≥150%	22.06	28.84
Employment status, %†		
Employed	75.29	78.14
Unemployed	2.67	3.73
Economically inactive	22.03	18.13
Immigration background, %†	17.16	17.09
Self‐reported diseases, %†		
Myocardial infarction	0.64	2.76
Angina pectoris	1.35	3.87
Heart failure	2.18	3.00
Arrythmias	9.48	8.82
Intermittent claudication	1.53	2.11
Hypertension	24.35	30.73
Diabetes	5.36	6.70
Hyperlipidemia	22.20	25.39
Stroke	1.21	1.90
BP, mm Hg†		
SBP, mean±SD	124.07±16.89	132.20±15.29
DBP, mean±SD	77.26±9.80	80.71±9.92
SBP/DBP ≥140/90 mm Hg, %	20.19	32.02
Anthropometric measurements, mean±SD or median (IQR)		
Waist circumference, cm||	85.77±13.57	96.63±12.91
Body mass index, kg/m^2^ †	24.9 (22.20–28.90)	26.60 (24.20–29.60)
Body fat, %‡	36.39±7.89	25.67±7.16
BMI categories, %†		
Underweight	1.69	0.45
Normal weight	48.76	32.03
Overweight	28.97	44.86
Obesity	20.59	22.66
Smoking status, %||		
Current	18.87	22.13
Former	30.44	36.18
Never	50.69	41.68
Alcohol consumption, %||		
Current (AUDIT‐C score >3 in women, >4 in men)	31.63	39.32
Current (AUDIT‐C score ≤3 in women, ≤4 in men)	58.82	53.00
Former	3.99	4.53
Never	5.56	3.15
Medication intake, %|| ^,^ ¶		
Antihypertensive therapy#	21.82	26.61
β‐Blockers	10.69	11.74
Biomarkers, mean±SD or median (IQR)		
Total cholesterol, mmol/L||	5.41±1.08	5.24±1.07
LDL cholesterol, mmol/L‡ ^,^ **	3.26±0.92	3.33±0.90
HDL cholesterol, mmol/L||	1.73±0.43	1.35±0.35
Triglycerides, mmol/L†† ^,^ ‡‡	1.22 (0.89–1.74)	1.61 (1.11–2.40)
Glycated hemoglobin, mmol/mol||	35.00 (33.00–38.00)	36.00 (33.00–39.00)
High‐sensitivity C‐reactive protein, mg/L‡‡ ^,^ §§	1.09 (0.53–2.64)	0.96 (0.51–2.06)
Family history of MI, %‡‡		
<60 y	9.12	8.44

AUDIT‐C, Alcohol Use Disorders Identification Test‐C; BMI indicates body mass index; BP, blood pressure; DBP, diastolic blood pressure; HDL, high‐density lipoprotein; IQR, interquartile range; LDL, low‐density lipoprotein; MI, myocardial infarction; and SBP, systolic blood pressure.

* A detailed description of missing data is presented in Table S2.

^†^ <1% is missing.

^‡^ 5%–10% are missing.

^§^ There were 4261 and 19 participants enrolled in vocational training and full‐time school, respectively.

^||^ 1%–5% are missing.

^¶^ According to the *Anatomical Therapeutic Chemical* (*ATC*) classification codes.

^#^ *ATC* codes C02, C03, and C07 to C09.

** LDL cholesterol is missing in 1 study center.

^††^ Triglycerides are missing in 2 study centers.

^‡‡^ ≥10% are missing.

^§§^ High‐sensitivity C‐reactive protein is missing in 5 study centers.

### Sex Differences in the Relationship of SEP With CVD and Risk Factors

**Figure 1:**
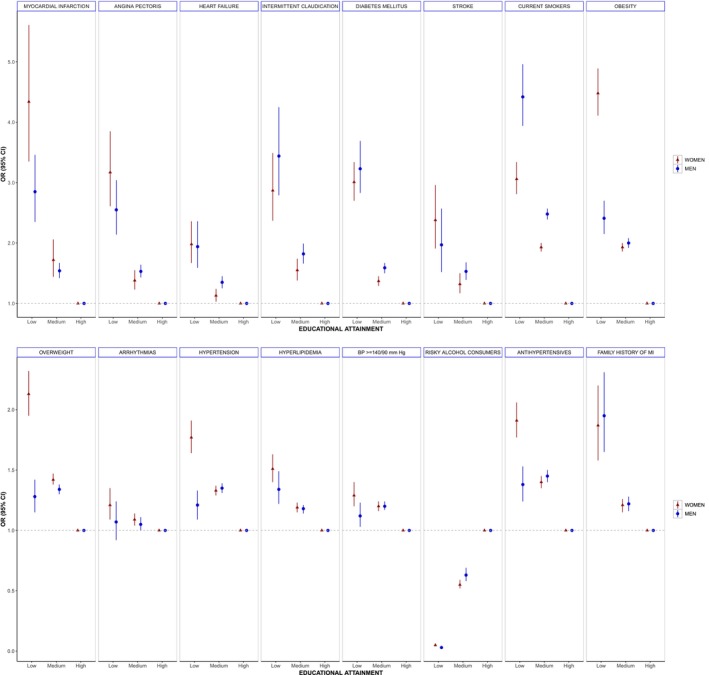
Associations between educational attainment and CVD or CVD risk factors in the NAKO study baseline assessment in women and men (n=200 279, 50.5% women, 49.5% men). Presented are age‐adjusted ORs with 95% CIs for low and middle education (reference is high education) from logistic or multinomial regression models. Analysis is based on the entire population at baseline. Reference categories in the multinomial logistic regression models are for BMI categories (normal weight), smoking status (never), and alcohol consumption (never). 95% CI for low vs high education in risky alcohol consumption is not visible due to sample size. BMI indicates body mass index; CVD, cardiovascular disease; NAKO, German National Cohort; and OR, odds ratio.

**Figure 2:**
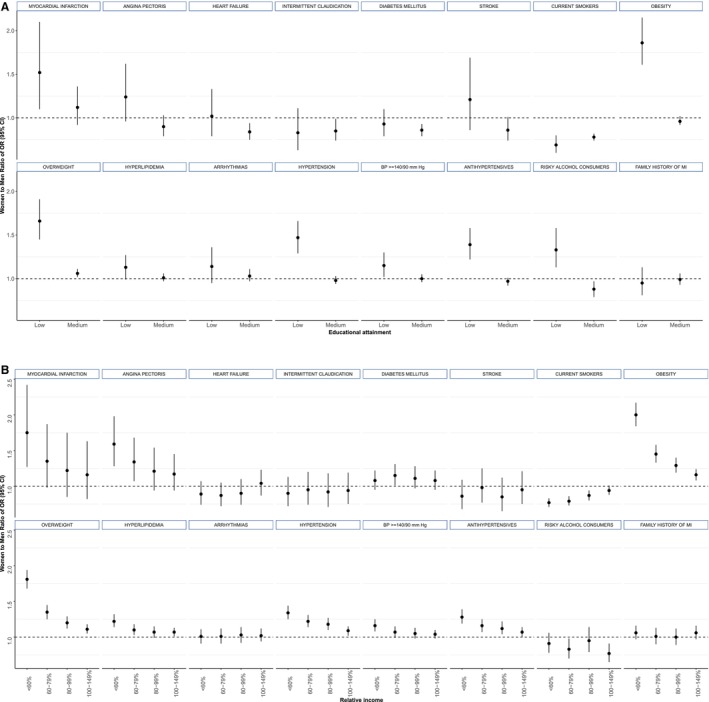
Women‐to‐men ratio of ORs with 95% CIs for CVD or CVD risk factors in the NAKO‐baseline assessment according to educational attainment and relative income. **A**, Educational attainment (reference is high education). **B**, Relative income (reference is relative income >150%). Analysis is based on the entire population at baseline. Reference categories in the multinomial logistic regression models are BMI categories (normal weight), smoking status (never), and alcohol consumption (never). Study participants are low education (n=3993 women, n=2462 men), middle education (n=46 777 women, n=37 470 men), and high education (n=50 302 women, n=59 275 men). Study participants are household income <60% (n=15 087 women, n=12 999 men), 60% to 79% (n=16 315 women, n=12 687 men), 80% to 99% (n=15 686 women, n=13 267 men), 100% to 149% (n=31 375 women, n=31 321 men), and >150% (n=22 609 women, n=28 934 men). Numbers correspond to the average estimates across all of the imputations. BP indicates blood pressure; CVD, cardiovascular disease; MI, myocardial infarction; NAKO, German National Cohort; and OR, odds ratio.

Overall, both women and men presented an inverse gradient between educational attainment and prevalent diseases and cardiovascular risk factors, with higher point estimates seen in lower versus higher educated women (Figure 1 and Table S3). Low versus high educational attainment was more strongly associated with MI, hypertension, blood pressure values ≥140/90 mm Hg, antihypertensive therapy, overweight, obesity, and current alcohol consumption in women compared with men (Figure 2 and Table S3). For example, compared with high education, women with low education had >4 times the odds of obesity (OR, 4.48 [95% CI, 4.11–4.89]) when compared with normal weight, almost twice the OR as observed in men (2.41 [95% CI, 2.15–2.70]; ROR, 1.86 [95% CI, 1.61–2.15]) (Figure 2). Conversely, women, compared with men were less likely to be current and former smokers than never smokers in the low educational category versus the high category. Adjusting for migration status did not change the point estimates of the sex‐specific associations considerably (Table S4).

Women with low versus high education had higher SBP and anthropometric measurements. In men, these differences between low versus high education were less pronounced (Table 2). An excess in the difference between low versus high education was observed to the detriment of women (as compared with men) for SBP, waist circumference, BMI, body fat, and low‐density lipoprotein cholesterol, with the highest differences in SBP (βwomen–βmen, 3.04 mm Hg [95% CI, 2.21–3.88]) and waist circumference (βwomen–βmen, 3.66 cm [95% CI, 2.96–4.35]). Women with low versus high education were associated with slightly lower values for HDL cholesterol and glycated hemoglobin than the equivalent for men. Evidence of sex differences for low education associated with biomarkers remained after exclusion of study centers with solely decentralized laboratory measurements (data not shown). Correction for multiple comparison did not change our interpretation of the observed sex differences for low versus high educational level (Table S5). However, for intermittent claudication, sex differences in middle versus high education did not meet the threshold established for multiple testing.

**Table 2:** Associations Between Education and Several Cardiovascular Risk Factors in the German National Cohort Study Baseline Assessment*

Cardiovascular risk factor	Women, N=101 071			Men, N=99 208			Women‐to‐men differences (95% CI)	
	Low education β (95% CI)	Middle education β (95% CI)	High education	Low education β (95% CI)	Middle education β (95% CI)	High education	Low education β_women_–β_men_ (95% CI)	Middle education β_women_–β_men_ (95% CI)
BP, mm Hg								
Systolic BP	2.50 (1.98 to 3.02)	1.93 (1.73 to 2.13)	Reference	−0.54 (−1.19 to 0.10)	1.33 (1.13 to 1.52)	Reference	3.04 (2.21 to 3.88)	0.60 (0.32 to 0.88)
Diastolic BP	0.69 (0.37 to 1.02)	0.97 (0.84 to 1.09)	Reference	0.19 (−0.22 to 0.59)	0.84 (0.71 to 0.97)	Reference	0.51 (−0.01 to 1.02)	0.12 (−0.05 to 0.30)
Anthropometric measurements								
Waist circumference, cm	7.37 (6.93 to 7.81)	3.06 (2.89 to 3.23)	Reference	3.72 (3.18 to 4.24)	2.90 (2.74 to 3.06)	Reference	3.66 (2.96 to 4.35)	0.16 (−0.07 to 0.39)
Body mass index, kg/m^2^ ‡	1.13 (1.11 to 1.13)	1.05 (1.05 to 1.05)	Reference	1.05 (1.04 to 1.05)	1.04 (1.03 to 1.04)	Reference	0.07 (0.06 to 0.08)	0.01 (0.01 to 0.02)
Body fat, %	4.37 (4.14 to 4.62)	1.97 (1.88 to 2.06)	Reference	2.49 (2.20 to 2.76)	1.39 (1.29 to 1.48)	Reference	1.89 (1.51 to 2.26)	0.59 (0.46 to 0.71)
Biomarkers								
Total cholesterol, mmol/L	−0.05 (−0.08 to 0.01)	0.02 (0.01 to 0.03)	Reference	−0.08 (−0.12 to −0.03)	0.04 (0.02 to 0.05)	Reference	0.03 (−0.03 to 0.08)	−0.02 (−0.04 to 0.002)
LDL cholesterol, mmol/L†	0.05 (0.01 to 0.08)	0.04 (0.03 to 0.05)	Reference	−0.02 (0.05 to 0.03)	0.03 (0.02 to 0.05)	Reference	0.06 (0.01 to 0.11)	0.01 (−0.01 to 0.02)
HDL cholesterol, mmol/L	−0.19 (−0.20 to −0.17)	−0.05 (−0.05 to −0.04)	Reference	−0.13 (−0.15 to −0.12)	−0.04 (−0.04 to −0.03)	Reference	−0.05 (−0.07 to −0.03)	−0.01 (−0.02 to −0.005)
HbA1c, mmol/mol‡	1.08 (1.07 to 1.08)	1.02 (1.01 to 1.02)	Reference	1.09 (1.08 to 1.10)	1.02 (1.02 to 1.02)	Reference	−0.01 (−0.02 to −0.007)	−0.01 (−0.01 to 0.009)
Triglycerides, mmol/L‡ ^,^ §	1.16 (1.15 to 1.19)	1.05 (1.05 to 1.06)	Reference	1.14 (1.11 to 1.17)	1.07 (1.06 to 1.07)	Reference	0.03 (−0.004 to 0.06)	−0.01 (−0.02 to 0.001)
hs‐CRP, mg/L‡ ^,^ ||	1.67 (1.59 to 1.75)	1.26 (1.24 to 1.28)	Reference	1.60 (1.52 to 1.69)	1.25 (1.23 to 1.27)	Reference	0.04 (−0.03 to 0.12)	0.01 (−0.02 to 0.03)

Presented are age‐adjusted sex‐specific ß coefficients with 95% CIs from linear regression models and women‐to‐men slope differences. BP indicates blood pressure; HbA1c, glycated hemoglobin; HDL, high‐density lipoprotein; hs‐CRP, high‐sensitivity C‐reactive protein; and LDL, low‐density lipoprotein.

* Analysis is based on the entire population at baseline. β estimates represent arithmetic differences in low and medium educational levels vs high, except for body mass index, HbA1c, triglycerides, and hs‐CRP, in which estimates are interpreted as the ratio of the geometric mean of the outcome in low or middle education over the geometric mean of the outcome in high education.

^†^ Data are from 159 512 study participants.

^‡^ Analyzed at the log scale; estimates were back transformed.

^§^ Data are from 172 766 study participants.

^||^ Data are from 122 468 study participants.

Analyses using relative income as exposure yielded largely similar findings as those analyses using education as the exposure, with a few exceptions (Table S6, Figure S4). For relative income, a higher magnitude of excess likelihood in women was observed for angina pectoris. For risky alcohol consumption, no sex differences were observed for the lowest relative income category (Figure 2).

### Sex Differences in the Relationship of SEP With Estimated CVD Risk

Table 3 shows the distribution of selected CVD risk factors across SEP categories in study participants eligible for the calculation of the SCORE2 algorithm (n=143 019). Participants were on average 53 years of age, and in general, detrimental cardiovascular risk factors were more likely in those with low SEP. A similar distribution of CVD risk factors was seen in the nonimputed data (Table S7). In the subgroup of participants eligible for the SCORE2, sex differences in self‐reported CVD and related risk factors in relation to educational attainment mirrored those observed for the whole NAKO population, except for hyperlipidemia and diastolic blood pressure, which presented sex differences (Tables S8 and S9). Additionally, sex differences were no longer evident for underweight.

**Table 3:** Cardiovascular Disease Risk Factor Distribution Across Categories of Socioeconomic Position in the German National Cohort Study Sample Eligible for the Systemic Coronary Risk Estimation‐2 Algorithm*

SEP	N=143 019†		Age, y, mean±SD		Current smoker (%)		SBP, median (IQR)		HDL cholesterol, median (IQR)		Total cholesterol, median (IQR)	
	Women	Men	Women	Men	Women	Men	Women	Men	Women	Men	Women	Men
Educational attainment												
Low	2806	1487	55.94±8.27	52.76±8.25	31.75	41.81	126 (116–139)	130 (121–141)	1.57 (1.31–1.88)	1.20 (1.03–1.43)	5.60 (4.90–6.30)	5.30 (4.70–6.00)
Medium	34 618	25 359	54.59±8.14	53.52±8.03	21.40	28.06	125 (115–137)	132 (123–143)	1.70 (1.43–2.00)	1.30 (1.10–1.55)	5.59 (4.90–6.30)	5.46 (4.80–6.16)
High	36 478	42 271	53.61±8.06	53.85±8.28	14.59	16.44	122 (112–134)	131 (122–141)	1.74 (1.48–2.03)	1.34 (1.13–1.58)	5.50 (4.84–6.20)	5.40 (4.80–6.08)
Relative income												
<60%	9657	7425	54.93±8.34	54.68±8.41	26.96	35.31	125 (114–137)	131 (122–143)	1.62 (1.37–1.92)	1.27 (1.06–1.52)	5.60 (4.90–6.31)	5.40 (4.70–6.10)
60%–79%	11 581	8104	55.55±8.59	54.69±8.66	19.97	25.23	125 (115–138)	132 (123–144)	1.68 (1.42–1.98)	1.31 (1.10–1.55)	5.60 (4.91–6.30)	5.40 (4.80–6.10)
80%–99%	11 311	9041	53.74±8.38	52.68±8.21	19.48	23.59	124 (114–136)	132 (123–142)	1.70 (1.44–1.99)	1.31 (1.10–1.55)	5.50 (4.81–6.20)	5.40 (4.80–6.10)
100%–149%	23 466	22 348	53.61±7.97	53.37±8.16	16.90	19.35	123 (113–135)	132 (123–142)	1.73 (1.47–2.03)	1.32 (1.12–1.57)	5.50 (4.88–6.21)	5.40 (4.80–6.10)
≥150%	17 888	22 198	53.82±7.59	53.78±7.87	14.16	16.07	122 (112–134)	131 (122–141)	1.79 (1.51–2.08)	1.35 (1.14–1.60)	5.50 (4.89–6.20)	5.40 (4.80–6.09)

HDL indicates high‐density lipoprotein; IQR, interquartile range; SEP, socioeconomic position; and SBP, systolic blood pressure.

* Analysis is based on the population 40 to 69 y of age.

^†^ Numbers correspond to the average estimates across all the imputations.

**Figure 3:**
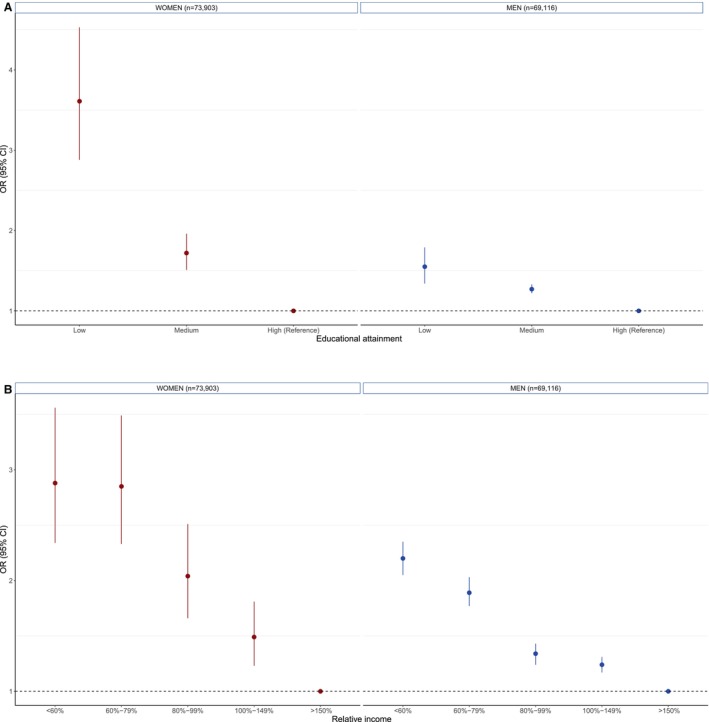
Sex‐specific OR with 95% CIs for a very high 10‐year risk of CVD in the NAKO‐baseline assessment according to educational attainment and relative income. **A**, Educational attainment. **B**, Relative income. Analysis is based on the population 40 to 69 years of age. Groups 40 to 49 and 50 to 59 years of age were merged. CVD indicates cardiovascular disease; NAKO, German National Cohort; and OR, odds ratio.

**Figure 4:**
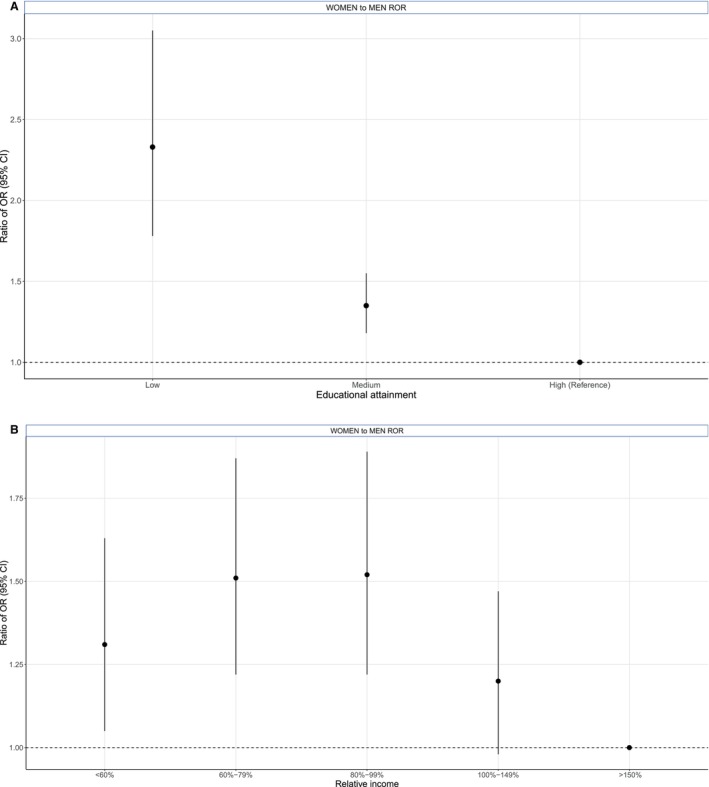
Women‐to‐men ratio of ORs with 95% CIs for very high 10‐year risk of CVD in the NAKO‐baseline assessment according to educational attainment and relative income. **A**, Educational attainment. **B**, Relative income. Analysis is based on the population 40 to 69 years of age. CVD indicates cardiovascular disease; NAKO, German National Cohort; OR, odds ratio; and ROR, ratio of ORs.

The proportion of a very high predicted 10‐year risk for CVD was lower in women than men: 0.07% versus 4.22% (age group 40–49 years) and 2.68% versus 23.00% (age group 50–69 years) (Figure S5). In women and men, there was a graded inverse relationship between educational attainment and a very high 10‐year CVD risk (Figure 3), but this inverse relationship was stronger in women compared with men. The OR for a very high risk for the lowest versus highest level of educational attainment was 3.61 (95% CI, 2.88–4.53) in women and 1.72 (95% CI, 1.51–1.96) in men, with a women‐to‐men ROR of 2.33 (95% CI, 1.78–3.05) (Figure 4). The OR for a very high 10‐year CVD risk for medium versus highest level of educational attainment was also higher in women, with a women‐to‐men ROR of 1.35 (95% CI, 1.18–1.55). Similar findings were observed for the association of relative income and very high SCORE2. The chance of having a very high 10‐year CVD risk was twice as high in individuals with low compared with those in the highest relative income group, with women‐to‐men ROR of 1.31 (95% CI, 1.05–1.63). Larger sex differences were observed in the relative income categories 60% to 79% and 80% to 99% compared with high income.

In sensitivity analysis stratifying for migration status, we observed a consistent inverse‐graded relationship between SEP and a very high 10‐year risk of CVD (Table S10). However, in the migrant group, the women‐to‐men difference in the middle versus high education categories was no longer apparent. Likewise, in the unemployed group, the excess likelihood for a very high 10‐year risk of CVD in women for the lowest versus highest educational level was not statistically significant. No sex differences in CVD risk were observed for the lowest versus highest income category, except for the economically inactive group.

### Sex Differences in Attenuations of Relationships Between SEP and Estimated CVD Risk by Adjustment for Risk Factors

We next adjusted the relationship between SEP and estimated risk CVD risk for age, smoking, HDL cholesterol, SBP, and antihypertensive treatment individually to quantify to what extent these risk factors statistically explain these relationships. In women, we observed an attenuation of the point estimates for the association of indicators of SEP and very high 10‐year risk of CVD after age adjustment, whereas in men, there was a stronger magnitude of the association for education and very high 10‐year CVD risk (Table 4). Therefore, sex differences were no longer present. In men, the association between education and very high 10‐year CVD risk was no longer statistically significant when adding smoking into the model. Furthermore, adjustments for antihypertensive treatment did not remove the sex differences. The 95% CI for the excess risk in women with relative income <60% versus ≥150% compared with men overlapped one in all adjusted models.

**Table 4:** Sex Differences in Attenuations of Relationships Between Socioeconomic Position and Estimated Cardiovascular Disease Risk by Adjustment for Risk Factors*

SEP	Women, OR (95% CI)	Men, OR (95% CI)	Women to men, ROR (95% CI)
Age adjusted			
N	73 903	69 116	
Educational attainment			
Low	2.48 (1.96–3.15)	2.25 (1.89–2.69)	1.10 (0.82–1.48)
Medium	1.45 (1.27–1.66)	1.53 (1.45–1.61)	0.95 (0.82–1.09)
High	Reference	Reference	Reference
Relative income			
<60%	2.05 (1.66–2.57)	2.17 (2.00–2.35)	0.94 (0.87–1.17)
60%–79%	1.70 (1.38–2.10)	1.69 (1.56; 1.83)	1.04 (0.90–1.19)
80%–99%	1.65 (1.34–2.05)	1.64 (1.51–1.78)	1.03 (0.90–1.19)
100%–149%	1.38 (1.13–1.69)	1.33 (1.24–1.41)	1.10 (0.98–1.23)
≥150%	Reference	Reference	Reference
Smoking‐adjusted			
Educational attainment			
Low	2.21 (1.75–2.79)	1.02 (0.88–1.17)	2.17 (1.65–2.85)
Medium	1.37 (1.20–1.56)	1.04 (0.99–1.09)	1.32 (1.15–1.51)
High	Reference	Reference	Reference
Relative income			
<60%	1.93 (1.56–2.39)	1.65 (1.54–1.78)	1.17 (0.93–1.47)
60%–79%	2.37 (1.93–2.91)	1.67 (1.55–1.79)	1.42 (1.14–1.76)
80%–99%	1.70 (1.38–2.10)	1.18 (1.10–1.27)	1.44 (1.15–1.80)
100%–149%	1.35 (1.11–1.65)	1.18 (1.11–1.25)	1.14 (0.93–1.41)
≥150%	Reference	Reference	Reference
SBP adjusted			
Educational attainment			
Low	2.77 (2.13–3.61)	1.79 (1.52–2.10)	1.55 (1.14–2.11)
Medium	1.50 (1.30–1.73)	1.21 (1.16–1.27)	1.24 (1.07–1.44)
High	Reference	Reference	Reference
Relative income			
<60%	2.50 (1.98–3.16)	2.31 (2.13–2.50)	1.08 (0.85–1.39)
60%–79%	2.33 (1.87–2.91)	1.90 (1.76–2.05)	1.23 (0.97–1.55)
80%–99%	1.85 (1.48–2.32)	1.32 (1.22–1.43)	1.40 (1.10–1.78)
100%–149%	1.42 (1.15–1.76)	1.24 (1.17–1.32)	1.15 (0.92–1.43)
≥150%	Reference	Reference	Reference
HDL adjusted			
Educational attainment			
Low	2.80 (2.22–3.54)	1.32 (1.14–1.53)	2.13 (1.61–2.82)
Medium	1.62 (1.42–1.84)	1.22 (1.17–1.28)	1.32 (1.15–1.51)
High	Reference	Reference	Reference
Relative income			
<60%	2.29 (1.85–2.83)	2.02 (1.88; 2.16)	1.13 (0.91–1.42)
60%–79%	2.48 (2.02–3.04)	1.82 (1.69–1.95)	1.37 (1.10–1.69)
80%–99%	1.82 (1.48–2.25)	1.28 (1.19–1.37)	1.43 (1.14–1.78)
100%–149%	1.39 (1.14–1.69)	1.21 (1.14–1.28)	1.15 (0.94–1.41)
≥150%	Reference	Reference	Reference
Antihypertensive medication adjusted			
Educational attainment			
Low	3.02 (2.40–3.80)	1.56 (1.35–1.81)	1.93 (1.46–2.55)
Medium	1.57 (1.38–1.79)	1.22 (1.17–1.28)	1.28 (1.12–1.47)
High	Reference	Reference	Reference
Relative income			
<60%	2.40 (1.95–2.97)	2.09 (1.95–2.24)	1.15 (0.92–1.43)
60%–79%	2.41 (1.97–2.96)	1.78 (1.66–1.91)	1.36 (1.10–1.68)
80%–99%	1.85 (1.50–2.29)	1.31 (1.22–1.40)	1.42 (1.14–1.77)
100%–149%	1.40 (1.15–1.70)	1.20 (1.14–1.27)	1.16 (0.95–1.42)
≥150%	Reference	Reference	Reference

Presented are sex‐specific adjusted ORs and women‐to‐men RORs with 95% CIs for very high 10‐y risk of cardiovascular disease in the German National Cohort study population eligible for the Systemic Coronary Risk Estimation‐2 risk algorithm. HDL indicates high‐density lipoprotein; OR, odds ratio; ROR, ratio of ORs; SEP, socioeconomic position; and SBP, systolic blood pressure.

* Analysis is based on the population 40 to 69 y of age.

### Use of Alternative Risk Scores

The distribution of risk factors in the populations when applying both the PCE and Reynolds Risk Score algorithms was similar to the one observed for the SCORE2 algorithm (Tables S11 through S15). Consistent with SCORE2 findings, in women and men there was an inverse relationship between education and high CVD risk (Table S16), albeit the association was not statistically significant in men using the Reynolds Risk Score. As for relative income, a similar inverse‐graded association with high 10‐year risk of CVD was observed, with higher point estimates in women. Age inclusion in the models yielded higher point estimates in men and attenuated the point estimates in women.

## DISCUSSION

In this large population‐based study, we found that in women compared with men, low SEP was more strongly associated with prevalent MI and hypertension, obesity, overweight, elevated blood pressure values, antihypertensive medication, and current alcohol consumption, but less strongly related to smoking (current and former). Furthermore, despite having a lower absolute CVD risk than men, women 40 to 69 years of age with low versus high SEP had higher odds of a very high 10‐year risk of first onset CVD than respective men. Our data suggest that although women and men share many of the traditional cardiovascular risk factors, the influence of low SEP on the risk of CVD over 10 years may differ between the sexes, to the detriment of women.

The present findings are in line with some studies that reported sex differences in the educational patterning of CVD‐related outcomes.^10^, ^25^ Similarly, a German population‐based study found that women with high versus low SEP (assessed using a multidimensional index) had 73% reduced odds of a high 10‐year CVD mortality risk, an association not observed in men.^26^ Although evidence exists on causal associations between low education and CHD,^27^ the underlying mechanisms for the excess likelihood in women observed in our study are unclear. A possible explanation for sex differences in SEP gradient and estimated 10‐year CVD risk could be the more pronounced social gradient for hypertension, obesity, and HDL cholesterol in women than in men. However, the stronger OR in women compared with men was attenuated but persisted after adjusting the association between education and very high 10‐year CVD risk for blood pressure, HDL cholesterol, or antihypertensive treatment, suggesting that these factors do not fully explain the sex differences. Alternatively, age is the strongest predictor of CVD risk models,^28^ and in the study sample considered for the SCORE2, older women were more likely to belong to the lower educational group, whereas in men, lower education was related to a slightly younger age. When age was included into the model examining the association between SEP and very high‐10‐year CVD risk, the social gradient remained, yet the magnitude of point estimates increased in men, decreased in women, and the excess likelihood associated with low education in women vanished. Furthermore, smoking‐adjusted models did not remove the excess likelihood in women in low education, but the association of education and high SCORE2 was no longer significant in men. In contrast, there was no evidence of sex difference in the association between relative income <60% (versus high) and a very high 10‐year CVD risk after adjustments. Taken together, our results suggest sex‐specific relations of risk factors on CVD risk scores across SEP and highlight variations in sex differences across different SEP indicators. Nevertheless, one may argue that the prediction algorithms include age, SBP, smoking, HDL cholesterol, and age interactions with these cardiovascular risk factors.

Speculatively, other potential explanations for the observed sex differences in high predicted CVD risk associated with low versus high SEP to the disadvantage of women may involve variations in risk factor management and treatment compliance. We observed a richer constellation of detrimental cardiovascular risk factors among women with low SEP. Women are less likely than men to receive guideline‐recommended preventive therapies, and research has suggested that German women at increased CVD risk may also be less aware of their risk.^29^, ^30^ In this context, consideration could be given to tailoring sex differences in health literacy among individuals with low SEP,^31^ given that CVD awareness campaigns have historically prioritized men. Marital status,^32^ diet,^33^ and environmental exposures^34^ may also differ across SEP, and may play a role in the observed associations for high CVD risk. Of note, adjustment for BMI did not remove the sex differences for high CVD risk (data not shown). In addition, socially disadvantaged people are commonly exposed to proinflammatory environments, such as psychosocial stress (financial instability, poor housing quality), insufficient or lack of access to health care providers, lack of safe environments to promote healthy behaviors (physical activity), and adverse health behaviors (smoking, poor nutrition).^35^, ^36^ Socioeconomic disadvantage has been proposed as an upstream determinant of increased low‐grade chronic inflammation through different mechanistic pathways.^37^, ^38^, ^39^ Specifically, lower SEP was linked to increased amygdalar activity and predicted major adverse cardiac events through increased sympathetic nervous system output with higher amygdalar activity, bone marrow activity, and arterial inflammation.^39^ Interestingly, allostatic load (stress hormones and response of cardiometabolic and inflammation systems to stress) has been shown as a mediator of the association between educational status and incidence of CHD, with the highest proportion mediated observed among women.^40^

The magnitude of the women‐to‐men differences for estimated CVD risk was larger in low education than low relative income categories. Education captures an individual's knowledge‐related resources,^9^ whereas relative income encompasses household income comparisons within a society. In contrast to income, which can fluctuate over time, education is typically acquired early in life and remains stable into adulthood. Therefore, educational attainment may reflect risks factors acquired earlier in life.^41^ SEP, however, is a complex multidimensional construct, and by using single indicators in our study, we might have underestimated its full effect on CVD.^14^ We have thus considered in sensitivity analyses another indicator of SEP, which yielded consistent results for the association between education and a very high 10‐year CVD risk. There was an exception for the unemployment group, which has been shown to have an independent effect on cardiovascular health.^42^ Of note, sex differences in cardiometabolic risk factors have been documented in individuals with and without migration background in a German population‐based study, with migrant women having higher BMI and glycated hemoglobin compared with men.^43^ In an attempt to consider other indicators of SEP not included in the SCORE2 algorithm (ie, family history of MI, ethnicity, treated SBP, high‐sensitivity C‐reactive protein), we replicated our analyses using other algorithms. Our findings for the graded relationship between SEP and high 10‐year CVD risk were consistent with the results from SCORE2, with higher point estimates in women and with an excess likelihood observed in women compared with men.

Men had a higher likelihood of very high 10‐year CVD risk than women. Thus, if women with the highest SEP indicator were chosen as the reference group, men in the lowest group of SEP were more likely to be in the very high 10‐year CVD risk group than women in the lowest SEP indicator group (Figure S6). Nevertheless, the CVD risk in women might be underestimated, because risk‐scores applied in this study relate to 10‐year rather than lifetime risk and might not be the most relevant time window for events in women.^2^

With >200 000 participants and a wide range of health indicators, the NAKO constitutes the largest epidemiological study conducted in Germany to date. Yet, our study has limitations. First, prevalent diseases are self‐reported, and it is uncertain whether the underreporting of the diseases we evaluated varies between sexes. Second, the presence of unknown/unmeasured confounders or mediators (eg, autoimmune inflammatory diseases more frequent in women, environmental factors) cannot be excluded. Third, because non‐European risk scores have been shown to overestimate the actual CVD risk in the German population,^44^ our findings using other algorithms should be interpreted with caution. We acknowledge that inclusion of ethnicity/social constructs in risk scores has been controversially debated, and its application in NAKO is challenging. However, we did not aim at risk model comparisons but to explore if our associations were consistent across different risk calculators. Fourth, exclusion of participants enrolled in education (0.05% of women and 0.09% of men 40–69 years of age were identified as having a very high CVD risk) is unlikely to alter our findings. Fifth, our cross‐sectional analyses preclude us from examining temporal relationships. Sixth, we cannot rule out the possibility that men living in socioeconomic disadvantage with an unfavorable CVD profile may have died prematurely, potentially leading to their underrepresentation in our analysis. Nonetheless, we observed a higher unfavorable cardiovascular profile (smoking status, low HDL cholesterol) among men in low SEP compared with those in high SEP, even when age was adjusted for. Seventh, participants of cohort studies tend to be more health conscious and may be less likely to have cardiovascular risk factors and less likely to belong to lower indicators of SEP compared with nonparticipants or to the general population.^45^ Although this may limit the generalizability of the estimated absolute risks, our primary focus was the relative comparison in risk factors and estimated disease risk between the sexes and the relation to social gradient, and men and women were recruited equally within age strata in the NAKO. Therefore, it is unlikely that our primary results are influenced by a healthy‐volunteer bias.

In conclusion, compared with those with high SEP, women with low SEP were more likely to have a detrimental cardiovascular risk profile than equivalent men. Although women had a substantially lower estimated 10‐year CVD risk compared with men, the likelihood of having a very high CVD risk associated with low versus high SEP was stronger in women than in men. Taken together, our findings suggest that CVD risk is more strongly influenced by SEP in women compared with men, which highlights the importance of social disparities on cardiovascular health and CVD prediction. Because women might be especially affected by the limitation of current risk prediction tools, initiatives to evaluate the inclusion of SEP, such as educational attainment, in the SCORE2 algorithm are urgently needed. From a public health perspective, our results underscore the importance of sex differences in the socioeconomic gradient along the CVD spectrum and may inform CVD preventive strategies and strengthen the implementation of individualized CVD risk‐based prevention efforts, ultimately improving cardiovascular health at the population level.

## Sources of Funding

This project was conducted with data (application number NAKO‐487) from the NAKO (www.nako.de). The NAKO is funded by the Federal Ministry of Education and Research (project funding reference numbers: 01ER1301A/B/C and 01ER1511D), federal states, and the Helmholtz Association, with additional financial support by the participating universities and the institutes of the Leibniz Association. S.A.E.P. is supported by a VIDI Fellowship from the Dutch Organization for Health Research and Development (ZonMw) (09150172010050).

## Disclosures

R.B.S. has received lecture fees and advisory board fees from Bristol Myers Squibb/Pfizer and Bayer outside this work. The remaining authors have no disclosures to report.

## Supporting information

Tables S1–S16
Figures S1–S6
