# Supplementary material for: Sex Differences in the Relationship of Socioeconomic Position With Cardiovascular Disease, Cardiovascular Risk Factors, and Estimated Cardiovascular Disease Risk: Results of the German National Cohort
Source: J Am Heart Assoc. 2025 Feb 25;14(5):e038708. doi: 10.1161/JAHA.124.038708 (PMC12132702; doi:10.1161/JAHA.124.038708)
Supplement: Supplementary file 1 — Tables S1–S16 Figures S1–S6 [file JAH3-14-e038708-s001.pdf]

Supplemental Material

Table S1. Definition of CVD and CVD risk factors

Cardiovascular disease or risk factor	Definition/measurements
Prevalent diseases	Self-reported medical history of myocardial infarction (MI), angina pectoris, heart failure, arrythmias, intermittent claudication, hypertension, diabetes mellitus, hyperlipidemia and stroke, all assessed through questionnaires (yes/no).
Smoking status and alcohol consumption	Categorized as current, former or never-smokers/consumers. Current alcohol consumption was further classified as risky if the Alcohol Use Disorders Identification Test (AUDIT-C) scored >4 in men or >3 in women, otherwise as non-risky
Antihypertensive medication intake	Assessed according to the Anatomical Therapeutic Chemical classification codes: C02, C03, C07, C08 and C09
Family history of MI	Self-reported family history of MI (maternal or paternal) diagnosed before the age of 60 years
Systolic (SBP)- and diastolic-blood pressure (DBP) (mmHg)	Measured twice in the sitting position after 5 min of rest and recorded at 2 min intervals using an Omron-Hem-705IT device, and the second values were used
Body mass index (BMI)	Calculated as body weight divided by the square of body height (kg/m²) and categorized as underweight (<18.5 kg/m²), normal weight (18.5-24.9 kg/m²), overweight (25.0-29.9 kg/m²) and obesity (≥30 kg/m²), respectively.<br>In n=4,627 (2.2%) study participants, weight and height were not measured. In these individuals BMI was calculated from self-reported weight and height.
Employment status	Categorized as employed, unemployed or economically inactive (i.e., pensioners).
Migration status	Defined as previously reported (yes/no). Migration status was recorded based on participants' nationality and country of birth and their parents' nationality and country of birth (reference provided in the manuscript).
Ethnicity	Categorized based on self-reported geographical regions of origin and phenotypic characteristics of the study participant.
Height (cm), weight (kg), waist circumference (WC) and body fat (%)	Measured with SECA (Stadiometer 274, mBCA 515, circumference measuring tape-201), Hamburg, Germany
Triglyceride, total-, low-density lipoprotein (LDL)- and high-density lipoprotein (HDL)-cholesterol concentrations (mmol/l)[a]	Measured photometrically in serum using either the Dimension Vista 1500 (Siemens Healthineers, Erlangen, Germany), the Advia 2400 (Siemens Healthineers, Erlangen, Germany), the Cobas 8000/8100/6000/701 systems (Roche Diagnostics, Rotkreuz, Schweiz), the AU680/5800/5822 systems (Beckman Coulter, Brea, USA) or the DxC 800 (Beckman Coulter, Brea, USA)
Glycated hemoglobin (HbA1c) concentrations (mmol/mol)[a]	Measured in EDTA-blood by high-performance liquid chromatography, immunoturbidimetry, immunoassay or capillary electrophoresis using either the Tosoh G8/G11 (Tosoh Bioscience, Inc., San Francisco, USA), the VariantTM II (Bio-Rad Laboratories, Hercules, USA), the DxC 800 (Beckman Coulter, Brea, USA), the Cobas c502/8000 systems (Roche Diagnostics, Rotkreuz, Schweiz), the Capillarys (Sebia, Lisses, Frankreich), the D-100 (Bio-Rad Laboratories, Hercules, USA), or the Dimension Vista (Siemens Healthineers, Erlangen, Germany),
High-sensitivity C-reactive protein (hs-CRP) concentrations (mg/l) [a]	Measured in serum by nephelometry or immunoturbidimetry using either the Dimension Vista 1500 (Siemens Healthineers, Erlangen, Germany) or the Cobas 8000 (Roche Diagnostics, Rotkreuz, Schweiz).

[a] Laboratory analyses were performed in decentral cooperating institutions close to the respective study centers. During the course of the baseline examination twelve of the eighteen study centers opted for central laboratory analyses at the Institute of Clinical Chemistry and Laboratory Medicine of the University Medicine Greifswald. Six study centers performed the analysis in external laboratories over the whole baseline period.

Table S2. Numbers and percentages of missing data in the NAKO study participants

	n (%) missing in All	n missing in Women	n missing in Men
**Missing < 1%**			
Sex[a]	0	-	-
Age[a]	0	-	-
Employment status[a]	1,644 (0.8)	821	823
Immigration background[a]	182 (0.08)	15	92
Myocardial infarction[a]	549 (0.3)	219	330
Angina pectoris[a]	1,123 (0.5)	442	681
Heart failure[a]	1,464 (0.7)	627	837
Arrythmias[a]	1,574 (0.8)	760	814
Intermittent claudication[a]	1,112 (0.5)	550	562
Hypertension[a]	923 (0.4)	365	558
Diabetes mellitus[a]	591 (0.3)	265	326
Hyperlipidemia[a]	2,123 (1.0)	863	1,260
Stroke[a]	701 (0.3)	341	360
Systolic blood pressure (SBP) [a]	483 (0.2)	296	187
Diastolic blood pressure (DBP) [a]	483 (0.2)	296	187
SBP/DBP ≥ 140/90 mmHg[a]	482 (0.2)	295	187
Body mass index[a]	1,014 (0.5)	532	482
BMI categories[a]	1,014 (0.5)	532	482
**Missing 1% to <5%**			
Waist circumference[a]	9,925 (4.8)	5,269	4,656
Smoking status[a]	8,894 (4.3)	4,723	4,171
Alcohol consumption[a]	9,691 (4.7)	5,147	4,544
Antihypertensive therapy[a]	3,139 (1.5)	1,534	1,605
Beta-blockers[a]	3,139 (1.5)	1,534	1,605
Total cholesterol [a]	8,801 (4.3)	5,045	3,756
HDL-cholesterol[a]	9,561 (4.7)	5,455	4,106
Glycated hemoglobin (HbA1c) [a]	9,614 (4.7)	5,495	4,119
**Missing 5% to <10%**			
Educational attainment[a]	14,436 (7.0)	7,440	6,996
Monthly net equivalent income[a]	13,951 (6.8)	8,089	5,862
Relative income[a]	13,951 (6.8)	8,089	5,862
Ethnicity/Country of origin	20,150 (9.8)	11,053	9,097
Body fat percentage[a]	13,915 (6.8)	7,429	6,486
LDL-cholesterol	19,986 (9.8)	10,749	9,237
**Missing ≥ 10%**			
Triglycerides	32,014 (15.6)	16,881	15,133
High-sensitivity C-reactive protein (hs-CRP)	82,312 (40.2)	42,446	39,866
Family history of MI[a]	33,029 (16.1)	17,510	15,519

LDL-cholesterol missing in one study center. Triglycerides missing in two study centers. Hs-CRP missing in 5 study centers.
[a]Variables included in MICE. Auxiliary variables considered: self-reported multiple sclerosis, rheumatoid arthritis, systemic lupus erythematosus, psoriasis and depression (missing <1% in each).

Table S3. Associations between educational attainment and CVD or CVD risk factors in the NAKO study-baseline assessment in women and men (n=200,279, 50.5% women, 49.5% men)*. Presented are age-adjusted odds ratios (OR) and women to men ratio of OR (ROR) with 95% CI for low and middle education (reference=high education) from logistic or multinomial regression models

	Women n=101,071			Men n=99,208			Women to Men ROR (95%CI)	
**Cardiovascular risk factor/CVD**	**Low education OR (95% CI)**	**Middle education OR (95% CI)**	**High education OR (95% CI)**	**Low education OR (95% CI)**	**Middle education OR (95% CI)**	**High education OR (95% CI)**	**Low education ROR (95% CI)**	**Middle education ROR (95% CI)**
**Self-reported diseases**								
Myocardial infarction	4.34 (3.35;5.61)	1.72 (1.44;2.06)	Reference	2.85 (2.35;3.46)	1.54 (1.42;1.67)	Reference	1.52 (1.10;2.10)	1.12 (0.92;1.36)
Angina pectoris	3.17 (2.61;3.85)	1.38 (1.23;1.55)	Reference	2.55 (2.14;3.04)	1.53 (1.43;1.64)	Reference	1.24 (0.96;1.62)	0.90 (0.79;1.03)
Heart Failure	1.98 (1.67;2.36)	1.13 (1.03;1.24)	Reference	1.94 (1.59;2.36)	1.35 (1.25;1.45)	Reference	1.02 (0.79;1.33)	0.84 (0.75;0.94)
Arrythmias	1.21 (1.09;1.35)	1.09 (1.04;1.14)	Reference	1.07 (0.92;1.24)	1.05 (1.00;1.11)	Reference	1.14 (0.95;1.36)	1.03 (0.97;1.11)
Intermittent claudication	2.87 (2.37;3.49)	1.55 (1.38;1.74)	Reference	3.44 (2.79;4.25)	1.82 (1.66;1.99)	Reference	0.83 (0.63;1.11)	0.85 (0.74;0.99)
Hypertension	1.77 (1.64;1.91)	1.33 (1.29;1.37)	Reference	1.21 (1.09;1.33)	1.35 (1.31,1.39)	Reference	1.47 (1.29;1.66)	0.98 (0.94;1.03)
Diabetes mellitus	3.01 (2.70;3.34)	1.37 (1.29;1.45)	Reference	3.23 (2.83;3.69)	1.59 (1.50;1.67)	Reference	0.93(0.79;1.10)	0.86 (0.79;0.93)
Hyperlipidemia	1.51 (1.40;1.63)	1.19 (1.15;1.23)	Reference	1.34 (1.22;1.49)	1.18 (1.14;1.21)	Reference	1.13 (0.99;1.27)	1.01 (0.97;1.06)
Stroke	2.38 (1.91;2.96)	1.32 (1.17;1.50)	Reference	1.97 (1.52;2.57)	1.53 (1.39;1.68)	Reference	1.21 (0.86;1.69)	0.86 (0.74;1.01)
**BMI categories**								
Normal weight	Reference	Reference	Reference	Reference	Reference	Reference	Reference	Reference
Underweight	1.64 (1.23;2.18)	0.79 (0.71;0.88)	Reference	3.76 (2.34;6.03)	2.15 (1.75;2.66)	Reference	0.44 (0.25;0.76)	0.37 (0.29;0.47)
Overweight	2.13 (1.95;2.32)	1.42 (1.38;1.47)	Reference	1.28 (1.15;1.42)	1.34 (1.30;1.38)	Reference	1.66 (1.45;1.91)	1.06 (1.02;1.11)
Obesity	4.48 (4.11;4.89)	1.93 (1.86;2.00)	Reference	2.41 (2.15;2.70)	2.00 (1.92;2.08)	Reference	1.86 (1.61;2.15)	0.96 (0.92;1.02)
**Blood pressure, ≥140/90 mmHg**	1.29 (1.20;1.40)	1.20 (1.16;1.24)	Reference	1.12 (1.03;1.23)	1.20 (1.17;1.24)	Reference	1.15 (1.02;1.30)	1.00 (0.96;1.05)
**Smoking status**								
Never	Reference	Reference	Reference	Reference	Reference	Reference	Reference	Reference
Former	1.05 (0.96;1.15)	1.30 (1.27;1.34)	Reference	1.50 (1.33;1.70)	1.59 (1.53;1.64)	Reference	0.70 (0.60;0.81)	0.82 (0.79;0.86)
Current	3.06 (2.81;3.34)	1.93 (1.86;2.00)	Reference	4.42 (3.94;4.96)	2.48 (2.39;2.57)	Reference	0.69 (0.60;0.80)	0.78 (0.74;0.82)
**Alcohol consumption**								
Never	Reference	Reference	Reference	Reference	Reference	Reference	Reference	Reference
Former	0.18 (0.15;0.21)	0.87 (0.79;0.95)	Reference	0.20 (0.17;0.24)	1.22 (1.10;1.35)	Reference	0.89 (0.71;1.11)	0.71 (0.62;0.82)
Current (AUDIT-C score >3 in women, >4 in men)	0.05 (0.04;0.05)	0.55 (0.52;0.59)	Reference	0.03 (0.03;0.04)	0.63 (0.58;0.69)	Reference	1.33 (1.13;1.58)	0.88 (0.79;0.97)
Current (AUDIT-C score ≤3 in women, ≤4 in men	0.06 (0.05;0.06)	0.64 (0.60;0.68)	Reference	0.04 (0.03;0.04)	0.60 (0.55;0.65)	Reference	1.54 (1.33;1.79)	1.07 (0.97;1.18)
**Medication intake**								
Antihypertensive therapy	1.91 (1.77;2.06)	1.40 (1.35;1.45)	Reference	1.38 (1.24;1.53)	1.45 (1.40;1.50)	Reference	1.39 (1.22;1.58)	0.97 (0.92;1.01)
Beta-blockers	1.70 (1.55;1.87)	1.35 (1.29;1.41)	Reference	1.62 (1.42;1.84)	1.49 (1.43;1.55)	Reference	1.05 (0.90;1.23)	0.91 (0.85;0.96)
**Family history of MI**								
<60 years	1.87 (1.58;2.20)	1.21 (1.15;1.26)	Reference	1.95 (1.65;2.31)	1.22 (1.16;1.28)	Reference	0.95 (0.81;1.13)	0.99 (0.93;1.06)

*Analysis based on the entire population at baseline. BMI, body mass index; CVD, cardiovascular disease; MI, myocardial infarction.
Models adjusted for age.

Table S4. Associations between educational attainment and CVD or CVD risk factors in the NAKO study-baseline assessment in women and men (n=200,279, 50.5% women, 49.5% men)*. Presented are age and migration status adjusted odds ratios (OR) with 95% CI for low and middle education (reference=high education) from logistic or multinomial regression models

	**Women N=101,071**		**Men N=99,208**	
	**Model 2**		**Model 2**	
**Cardiovascular risk factor/CVD**	**Low education OR (95% CI)**	**Middle education OR (95% CI)**	**Low education OR (95% CI)**	**Middle education OR (95% CI)**
**Self-reported diseases**				
Myocardial infarction	4.23 (3.26;5.48)	1.72 (1.44;2.06)	2.81 (2.30;3.43)	1.54 (1.42;1.67)
Angina pectoris	3.03 (2.49;3.68)	1.39 (1.23;1.56)	2.51 (2.10;3.00)	1.53 (1.43;1.64)
Heart Failure	1.94 (1.63;2.31)	1.13 (1.03;1.24)	1.98 (1.62;2.42)	1.35 (1.25;1.45)
Arrythmias	1.24 (1.11;1.38)	1.09 (1.04;1.14)	1.13 (0.97;1.32)	1.05 (1.01;1.11)
Intermittent claudication	2.87 (2.36;3.49)	1.55 (1.38;1.74)	3.66 (2.94;4.54)	1.82 (1.66;2.00)
Hypertension	1.79 (1.66;1.93)	1.33 (1.29;1.37)	1.30 (1.17;1.43)	1.35 (1.31,1.40)
Diabetes mellitus	2.84 (2.55;3.16)	1.37 (1.29;1.45)	3.05 (2.66;3.49)	1.58 (1.50;1.67)
Hyperlipidemia	1.45 (1.34;1.57)	1.20 (1.16;1.24)	1.33 (1.20;1.47)	1.18 (1.14;1.21)
Stroke	2.44 (1.96;3.03)	1.32 (1.17;1.50)	2.06 (1.58;2.70)	1.53 (1.40;1.69)
**BMI categories**				
Normal weight	Reference	Reference		Reference
Underweight	1.58 (1.18;2.12)	0.79 (0.71;0.89)	3.47 (2.13;5.66)	2.15 (1.74;2.65)
Overweight	2.06 (1.88;2.25)	1.43 (1.38;1.47)	1.18 (1.06;1.32)	1.34 (1.29;1.38)
Obesity	4.34 (3.98;4.74)	1.93 (1.86;2.00)	2.24 (1.99;2.52)	1.99 (1.92;2.07)
**Blood pressure, ≥140/90 mm Hg**	1.32 (1.22;1.43)	1.20 (1.16;1.24)	1.20 (1.09;1.32)	1.21 (1.17;1.24)
**Smoking status**				
Never	Reference			Reference
Former	1.11 (1.01;1.21)	1.30 (1.26;1.34)	1.49 (1.31;1.69)	1.59 (1.53;1.64)
Current	3.15 (2.89;3.44)	1.93 (1.86;2.00)	4.32 (3.85;4.85)	2.48 (2.39;2.57)
**Alcohol consumption**				
Never	Reference			Reference
Former	0.23 (0.20;0.27)	0.84 (0.77;0.92)	0.30 (0.25;0.35)	1.24 (1.12;1.37)
Current (AUDIT-C score >3 in women, >4 in men)	0.06 (0.06;0.07)	0.53 (0.50;0.57)	0.06 (0.05;0.07)	0.65 (0.60;0.71)
Current (AUDIT-C score ≤3 in women, ≤4 in men	0.08 (0.07;0.08)	0.62 (0.58;0.66)	0.06 (0.05;0.07)	0.61 (0.56;0.66)
**Medication intake**				
Antihypertensive therapy	1.94 (1.79;2.10)	1.40 (1.35;1.45)	1.48 (1.33;1.64)	1.46 (1.41;1.50)
Beta-blockers	1.71 (1.50;1.95)	1.49 (1.43;1.56)	1.71 (1.50;1.95)	1.49 (1.43;1.56)
**Maternal/paternal history of MI**				
<60 years	1.74 (1.48;2.06)	1.21 (1.16;1.27)	1.84 (1.56;2.17)	1.21 (1.16;1.28)

*Analysis based on the entire population at baseline. BMI: body mass index; CVD: cardiovascular disease; MI: myocardial infarction
Model 2: adjusted for age + migration status

Table S5. Associations between educational attainment and CVD or CVD risk factors in the NAKO study-baseline assessment (n=200,279, 50.5% women, 49.5% men)*. Presented are age-adjusted women to men ratio of OR (ROR) with 95% CI and βwomen- βmen (95%CI) differences for low and middle education (reference=high education) from logistic or multinomial regression models, with p-value corrected by the false discovery rate method

	Women to Men ROR (95%CI) Low education			Women to Men ROR (95%CI) Middle education		
**Cardiovascular risk factor/CVD**	**ROR (95% CI)**	**p-value**	**Corrected p-value (FDR)**	**ROR (95% CI)**	**p-value**	**Corrected p-value (FDR)**
**Self-reported diseases**						
Myocardial infarction	1.52 (1.10;2.10)	0.01	0.02	1.12 (0.92;1.36)	0.28	0.35
Angina pectoris	1.24 (0.96;1.62)	0.09	0.16	0.90 (0.79;1.03)	0.13	0.21
Heart Failure	1.02 (0.79;1.33)	0.87	0.88	0.84 (0.75;0.94)	0.003	0.007
Arrythmias	1.14 (0.95;1.36)	0.17	0.25	1.03 (0.97;1.11)	0.32	0.38
Intermittent claudication	0.83 (0.63;1.11)	0.21	0.27	0.85 (0.74;0.99)	0.04	0.07
Hypertension	1.47 (1.29;1.66)	<0.01	<0.01	0.98 (0.94;1.03)	0.50	0.56
Diabetes mellitus	0.93(0.79;1.10)	0.40	0.46	0.86 (0.79;0.93)	<0.01	<0.01
Hyperlipidemia	1.13 (0.99;1.27)	0.06	0.12	1.01 (0.97;1.06)	0.55	0.58
Stroke	1.21 (0.86;1.69)	0.27	0.35	0.86 (0.74;1.01)	0.07	0.11
**BMI categories**						
Normal weight	Reference			Reference		
Underweight	0.44 (0.25;0.76)	<0.01	<0.01	0.37 (0.29;0.47)	<0.01	<0.01
Overweight	1.66 (1.45;1.91)	<0.01	<0.01	1.06 (1.02;1.11)	<0.01	0.01
Obesity	1.86 (1.61;2.15)	<0.01	<0.01	0.96 (0.92;1.02)	0.18	0.25
**Blood pressure, ≥140/90 mmHg**	1.15 (1.02;1.30)	0.02	0.04	1.00 (0.96;1.05)	0.98	0.98
**Smoking status**						
Never	Reference			Reference		
Former	0.70 (0.60;0.81)	<0.01	<0.01	0.82 (0.79;0.86)	<0.01	<0.01
Current	0.69 (0.60;0.80)	<0.01	<0.01	0.78 (0.74;0.82)	<0.01	<0.01
**Alcohol consumption**						
Never	Reference			Reference		
Former	0.89 (0.71;1.11)	0.31	0.37	0.71 (0.62;0.82)	<0.01	<0.01
Current (AUDIT-C score >3 in women, >4 in men)	1.33 (1.13;1.58)	<0.01	<0.01	0.88 (0.79;0.97)	0.01	0.03
Current (AUDIT-C score ≤3 in women, ≤4 in men	1.54 (1.33;1.79)	<0.01	<0.01	1.07 (0.97;1.18)	0.19	0.26
**Medication intake**						
Antihypertensive therapy	1.39 (1.22;1.58)	<0.01	<0.01	0.97 (0.92;1.01)	0.17	0.25
Beta-blockers	1.05 (0.90;1.23)	0.54	0.59	0.91 (0.85;0.96)	<0.01	<0.01
**Family history of MI**						
<60 years	0.95 (0.81;1.13)	0.58	0.61	0.99 (0.93;1.06)	0.80	0.82
	**Women to Men differences βwomen- βmen (95%CI) Low education**			**Women to Men differences βwomen- βmen (95%CI) Middle education**		
**Blood pressure (BP), mmHg**						
Systolic BP	3.04 (2.21;3.88)	<0.01	<0.01	0.60 (0.32;0.88)	<0.01	<0.01

Diastolic BP	0.51 (-0.01;1.02)	0.06	0.11	0.12 (-0.05;0.30)	0.17	0.25
**Anthropometric measurements**						
Waist circumference (cm)	3.66 (2.96;4.35)	<0.01	<0.01	0.16 (-0.07;0.39)	0.18	0.25
Body mass index (kg/m^2)[2]	0.07 (0.06;0.08)	<0.01	<0.01	0.01 (0.01;0.02)	<0.01	<0.01
Body fat (%)	1.89 (1.51;2.26)	<0.01	<0.01	0.59 (0.46;0.71)	<0.01	<0.01
**Biomarkers:**						
Total cholesterol (mmol/l)	0.03 (-0.03;0.08)	0.33	0.39	-0.02 (-0.04;0.002)	0.08	0.13
LDL-cholesterol (mmol/l) [1]	0.06 (0.01;0.11)	0.01	0.03	0.01 (-0.01;0.02)	0.23	0.37
HDL-cholesterol (mmol/l)	-0.05 (-0.07;-0.03)	<0.01	<0.01	-0.01 (-0.02;-0.005)	<0.01	<0.01
HbA1c (mmol/mol) [2]	-0.01 (-0.02;-0.007)	<0.01	<0.01	-0.01 (-0.01;-0.007)	<0.01	<0.01
Triglycerides (mmol/l)[2,3]	0.03 (-0.004;0.06)	0.08	0.14	-0.01 (-0.02;0.001)	0.09	0.14
hs-CRP (mg/l)[2,4]	0.04 (-0.03;0.12)	0.27	0.35	0.01 (-0.02;0.03)	0.53	0.58

*Analysis based on the entire population at baseline. FDR, false discovery rate; BMI, body mass index; CVD, cardiovascular disease; MI, myocardial infarction.

Table S6. Associations between relative income and CVD or CVD risk factors in the NAKO study-baseline assessment in women and men (n=200,279, 50.5% women, 49.5% men)*. Presented are age-adjusted odds ratios (OR) and women to men ratio of OR (ROR) with 95% CI for low- and middle-income categories (reference=high income) from logistic or multinomial regression models

Cardiovascular risk factor/CVD	<60 % OR (95% CI)	60 % -79 % OR (95% CI)	80 % -99 % OR (95% CI)	100 % -149 % OR (95% CI)	≥150 % OR (95% CI)
			**Women n=101,071**		
**Self-reported diseases**					
Myocardial infarction	4.39 (3.27;5.89)	2.61 (1.93;3.51)	1.83 (1.31;2.55)	1.42 (1.03;1.95)	Reference
Angina pectoris	3.57 (2.94;4.34)	2.41 (1.98;2.94)	1.79 (1.44;2.22)	1.46 (1.20;1.78)	Reference
Heart Failure	2.34 (2.02;2.71)	1.77 (1.52;2.05)	1.42 (1.21;1.67)	1.35 (1.17;1.55)	Reference
Arrhythmias	1.30 (1.21;1.39)	1.14 (1.06;1.22)	1.13 (1.05;1.22)	1.07 (1.00;1.14)	Reference
Intermittent claudication	3.55 (2.99;4.22)	2.19 (1.81;2.63)	1.70 (1.39;2.07)	1.34 (1.12;1.61)	Reference
Hypertension	1.97 (1.87;2.07)	1.69 (1.61;1.79)	1.54(1.46;1.62)	1.34 (1.28;1.41)	Reference
Diabetes mellitus	3.44 (3.12;3.80)	2.46 (2.22;2.72)	1.90 (1.71;2.12)	1.50 (1.36;1.66)	Reference
Hyperlipidemia	1.39 (1.32;1.46)	1.21 (1.15;1.27)	1.15 (1.09;1.21)	1.10 (1.05;1.15)	Reference
Stroke	2.29 (1.89;2.78)	1.87 (1.54;2.26)	1.28 (1.03;1.59)	1.14 (0.94;1.38)	Reference
**BMI categories**					
Normal weight	Reference	Reference	Reference	Reference	Reference
Underweight	1.68 (1.44;1.97)	1.05 (0.88;1.26)	0.97 (0.82;1.15)	0.88 (0.76;1.02)	Reference
Overweight	1.71 (1.63;1.81)	1.55(1.47;1.63)	1.41(1.34;1.48)	1.31(1.26;1.37)	Reference
Obesity	3.69 (3.48;3.92)	2.56 (2.42;2.71)	2.12 (2.00;2.25)	1.65 (1.57;1.74)	Reference
**Blood pressure, ≥140/90 mm Hg**	1.26 (1.19;1.33)	1.19 (1.13;1.26)	1.21 (1.14;1.28)	1.13 (1.08;1.19)	Reference
**Smoking status**					
Never	Reference	Reference	Reference	Reference	Reference
Former	0.79 (0.75;0.83)	0.92 (0.87;0.96)	0.99 (0.95;1.04)	1.03 (0.99;1.08)	Reference
Current	2.03 (1.91;2.15)	1.54 (1.45;1.63)	1.46 (1.38;1.55)	1.25 (1.19;1.32)	Reference
**Alcohol consumption**					
Never	Reference	Reference	Reference	Reference	Reference
Former	0.47 (0.41;0.55)	0.85 (0.73;0.98)	0.92 (0.78;1.08)	1.04 (0.90;1.21)	Reference
Current (AUDIT-C score >3 in women, >4 in men)	0.10 (0.09;0.11)	0.27 (0.24;0.30)	0.40 (0.35;0.45)	0.58 (0.52;0.65)	Reference
Current (AUDIT-C score ≤3 in women, ≤4 in men	0.16 (0.15;0.18)	0.45 (0.40;0.50)	0.62 (0.55;0.70)	0.81 (0.73;0.90)	Reference
**Medication intake**					
Antihypertensive therapy	2.13 (2.02;2.26)	1.81 (1.71;1.91)	1.56 (1.48;1.66)	1.38 (1.31;1.45)	Reference
Beta-blockers	2.16 (2.01;2.33)	1.93 (1.79;2.08)	1.66 (1.54;1.79)	1.45 (1.36;1.56)	Reference
**Family history of MI**					
<60 years	1.34 (1.23;1.45)	1.11 (1.02;1.20)	1.01 (0.93;1.09)	1.03 (0.97;1.09)	Reference
			**Men n=99,208**		
**Self-reported diseases**					
Myocardial infarction	2.50 (2.21;2.83)	1.93 (1.70;2.19)	1.50 (1.30;1.73)	1.23 (1.09;1.38)	Reference
Angina pectoris	2.25 (2.03;2.49)	1.80 (1.62;2.00)	1.48 (1.31;1.67)	1.25(1.14;1.38)	Reference
Heart Failure	2.64 (2.35;2.95)	2.03 (1.81;2.29)	1.58 (1.38;1.80)	1.30 (1.17;1.45)	Reference
Arrhythmias	1.29 (1.20;1.39)	1.13 (1.05;1.22)	1.10 (1.02;1.19)	1.04 (0.98;1.10)	Reference
Intermittent claudication	3.95 (3.43;4.55)	2.31 (1.98;2.69)	1.85 (1.57;2.18)	1.43 (1.23;1.65)	Reference
Hypertension	1.47 (1.40;1.54)	1.39 (1.32;1.46)	1.30 (1.24;1.37)	1.24 (1.19;1.28)	Reference
Diabetes mellitus	3.20 (2.95;3.47)	2.13 (1.96;2.32)	1.71 (1.56;1.88)	1.40 (1.29;1.51)	Reference
Hyperlipidemia	1.13 (1.08;1.19)	1.10 (1.05;1.16)	1.08 (1.02;1.13)	1.03 (0.99;1.07)	Reference
Stroke	2.66 (2.31;3.06)	1.91 (1.64;2.21)	1.50 (1.27;1.77)	1.20 (1.05;1.38)	Reference
**BMI categories**					
Normal weight	Reference	Reference	Reference	Reference	Reference
Underweight	5.71 (4.07;8.01)	3.06 (2.10;4.46)	2.33 (1.55;3.51)	1.44 (1.00;2.09)	Reference
Overweight	0.95 (0.90;1.00)	1.15 (1.09;1.21)	1.17 (1.12;1.23)	1.18 (1.13;1.22)	Reference
Obesity	1.85 (1.74;1.96)	1.77 (1.66;1.87)	1.64 (1.55;1.75)	1.43 (1.36;1.49)	Reference
**Blood pressure, ≥140/90 mm Hg**	1.08 (1.03;1.13)	1.12 (1.07;1.17)	1.15 (1.10;1.21)	1.09 (1.05;1.13)	Reference
**Smoking status**					
Never	Reference	Reference	Reference	Reference	Reference
Former	1.10 (1.04;1.16)	1.23 (1.17;1.30)	1.16 (1.10;1.22)	1.14 (1.10;1.19)	Reference
Current	2.65 (2.50;2.80)	1.94 (1.83;2.06)	1.68 (1.59;1.77)	1.33 (1.27;1.39)	Reference
**Alcohol consumption**					
Never	Reference	Reference	Reference	Reference	Reference
Former	0.62 (0.53;0.72)	1.11 (0.94;1.30)	1.00 (0.84;1.19)	1.19 (1.01;1.39)	Reference

Current (AUDIT-C score >3 in women, >4 in men)	0.11 (0.10;0.13)	0.33 (0.29;0.38)	0.42 (0.36;0.48)	0.75 (0.66;0.86)	Reference
Current (AUDIT-C score ≤3 in women, ≤4 in men	0.13 (0.12;0.15)	0.38 (0.33;0.43)	0.50 (0.44;0.58)	0.84 (0.74;0.96)	Reference
**Medication intake**					
Antihypertensive therapy	1.66 (1.58;1.75)	1.56 (1.48;1.64)	1.39 (1.32;1.47)	1.29 (1.24;1.35)	Reference
Beta-blockers	2.01 (1.88;2.15)	1.74 (1.63;1.86)	1.53 (1.42;1.64)	1.32 (1.25;1.40)	Reference
**Family history of MI**					
<60 years	1.27 (1.16;1.39)	1.10 (1.01;1.18)	1.02 (0.94;1.10)	0.97 (0.91;1.03)	Reference
			**Women to Men ROR (95% CI)**		
**Self-reported diseases**					
Myocardial infarction	1.75 (1.27;2.42)	1.35 (0.98;1.87)	1.22 (0.85;1.75)	1.16 (0.82;1.63)	Reference
Angina pectoris	1.59 (1.28;1.98)	1.34 (1.07;1.68)	1.21 (0.94;1.54)	1.17 (0.94;1.45)	Reference
Heart Failure	0.89 (0.74;1.07)	0.87 (0.72;1.05)	0.90 (0.74;1.10)	1.04 (0.87;1.23)	Reference
Arrhythmias	1.01 (0.91;1.11)	1.01 (0.91;1.12)	1.03 (0.92;1.14)	1.02 (0.94;1.12)	Reference
Intermittent claudication	0.90 (0.72;1.13)	0.95 (0.74;1.20)	0.92 (0.71;1.18)	0.94 (0.75;1.19)	Reference
Hypertension	1.34 (1.25;1.44)	1.22 (1.14;1.31)	1.18 (1.10;1.27)	1.09 (1.02;1.15)	Reference
Diabetes mellitus	1.08 (0.95;1.22)	1.15 (1.01;1.31)	1.11 (0.97;1.28)	1.08 (0.95;1.22)	Reference
Hyperlipidemia	1.22 (1.14;1.32)	1.10 (1.03;1.18)	1.07 (0.99;1.15)	1.07 (1.01;1.13)	Reference
Stroke	0.86 (0.68;1.09)	0.98 (0.77;1.25)	0.85 (0.65;1.12)	0.95 (0.75;1.21)	Reference
**BMI categories**					
Normal weight	Reference	Reference	Reference	Reference	Reference
Underweight	0.30 (0.20;0.43)	0.34 (0.23;0.52)	0.42 (0.27;0.65)	0.61 (0.41;0.91)	Reference
Overweight	1.81 (1.68;1.94)	1.35 (1.25;1.45)	1.20 (1.12;1.29)	1.11 (1.05;1.18)	Reference
Obesity	2.00 (1.84;2.17)	1.45 (1.33;1.58)	1.29 (1.19;1.40)	1.16 (1.08;1.24)	Reference
**Blood pressure, ≥140/90 mm Hg**	1.16 (1.08;1.25)	1.07 (0.99;1.15)	1.05 (0.98;1.13)	1.04 (0.98;1.10)	Reference
**Smoking status**					
Never	Reference	Reference	Reference	Reference	Reference
Former	0.72 (0.67;0.77)	0.74 (0.69;0.80)	0.86 (0.80;0.92)	0.91 (0.86;0.96)	Reference
Current	0.77 (0.71;0.83)	0.79 (0.73;0.86)	0.87 (0.80;0.94)	0.94 (0.88;1.01)	Reference
**Alcohol consumption**					
Never	Reference	Reference	Reference	Reference	Reference
Former	0.76 (0.62;0.94)	0.76 (0.61;0.95)	0.92 (0.72;1.17)	0.88 (0.71;1.09)	Reference
Current (AUDIT-C score >3 in women, >4 in men)	0.91 (0.78;1.06)	0.83 (0.70;0.98)	0.95 (0.79;1.14)	0.77 (0.65;0.91)	Reference
Current (AUDIT-C score ≤3 in women, ≤4 in men	1.27 (1.09;1.48)	1.18 (0.99;1.39)	1.23 (1.03;1.48)	0.95 (0.81;1.13)	Reference
Antihypertensive therapy	1.28 (1.19;1.39)	1.16 (1.07;1.25)	1.12 (1.04;1.22)	1.07 (1.00;1.14)	Reference
Beta-blockers	1.08 (0.98;1.19)	1.11 (1.01;1.23)	1.09 (0.98;1.20)	1.10 (1.00;1.20)	Reference
Family history of MI <60 years	1.06 (0.97;1.16)	1.01 (0.90;1.13)	1.00 (0.89;1.12)	1.06 (0.97;1.16)	Reference

*Analysis based on the entire population at baseline. BMI, body mass index; CVD, cardiovascular disease; MI, myocardial infarction

Table S7. CVD risk factors distribution across categories of SES in the NAKO study population eligible for the SCORE2 risk algorithm* (non-imputed data)

	Age (mean, SD)		Current smoker (%)		SBP (median, IQR)		HDL-cholesterol (median, IQR)		Total cholesterol (median, IQR)	
	**Women**	**Men**	**Women**	**Men**	**Women**	**Men**	**Women**	**Men**	**Women**	**Men**
Educational attainment										
**Low** (n=1,785 W; 906 M)	56.51 (8.20)	52.67 (8.17)	31.82	42.27	127.00 (117.00-140.00)	130.00 (121.00-141.00)	1.61 (1.35-1.93)	1.22 (1.04-1.45)	5.70 (4.99-6.40)	5.30 (4.76-6.08)
**Medium** (n=29,004 W; 21,472 M)	54.42 (8.12)	53.34 (7.98)	21.07	27.78	125.00 (115.00-137.00)	132.00 (123.00-143.00)	1.70 (1.44-2.01)	1.30 (1.10-1.55)	5.56 (4.90-6.30)	5.44 (4.80-6.16)
**High** (n=30,998 W; 36,823 M)	53.48 (8.06)	53.77 (8.29)	14.57	16.19	122.00 (112.00-134.00)	131.00 (12.002-141.00)	1.74 (1.48-2.04)	1.34 (1.14-1.59)	5.50 (4.81-6.20)	5.40 (4.80-6.08)
Relative income										
**<60 % (**n=7,329 W; 5,803 M)	54.70 (8.30)	54.60 (8.40)	27.03	34.53	125.00 (114.00-137.00)	132.00 (122.00-143.00)	1.60 (1.40-1.90)	1.30 (1.10-1.50)	5.60 (4.90-6.30)	5.40 (4.70-6.10)
**60 %** -79 % (n=9,488 W; 6,942 M)	55.29 (8.59)	54.50 (8.65)	20.03	25.18	125.00 (115.00-137.00)	132.00 (123.00-143.00)	1.68 (1.42-1.98)	1.31 (1.10-1.55)	5.60 (4.90-6.30)	5.40 (4.80-6.10)
**80 % -99 %** (n=9,557 W; 8,003 M)	53.54 (8.38)	52.53 (8.18)	19.68	23.71	124.00 (113.00-136.00)	132.00 (123.00-142.00)	1.70 (1.43-1.99)	1.31 (1.10-1.55)	5.50 (4.80-6.20)	5.40 (4.80-6.10)
**100 % -149** % (n=20,040 W; 19,879 M)	53.45 (7.95)	53.22 (8.15)	17.14	19.23	123.00 (113.00-135.00)	132.00 (123.00-142.00)	1.72 (1.46-2.03)	1.32 (1.12-1.57)	5.50 (4.86-6.20)	5.40 (4.80-6.10)
**≥150 %** (n=15,324 W; 19,916 M)	53.68 (7.55)	53.64 (7.84)	14.29	15.88	122.00 (112.00-134.00)	131.00 (122.00-141.00)	1.81 (1.50-2.08)	1.35 (1.15-1.59)	5.50 (4.88-6.20)	5.40 (4.80-6.08)

*Analysis based on the population aged 40-69 years. M: Men, W: Women

Table S8. Associations between educational attainment and CVD or CVD risk factors in the NAKO study-baseline assessment in women and men 40-69 years old eligible for the SCORE2 algorithm (n=143,019, 51.7% women, 48.3% men)*. Presented are age-adjusted odds ratios (OR) and women to men ratio of OR (ROR) with 95% CI for low and middle education (reference=high education) from logistic or multinomial regression models

	Women N=73,903			Men N=69,116			Women to Men ROR (95%CI)	
**Cardiovascular risk factor/CVD**	**Low education OR (95% CI)**	**Middle education OR (95% CI)**	**High education OR (95% CI)**	**Low education OR (95% CI)**	**Middle education OR (95% CI)**	**High education OR (95% CI)**	**Low education ROR (95% CI)**	**Middle education ROR (95% CI)**
**Self-reported diseases**								
Angina pectoris	2.80 (2.12;3.69)	1.19 (1.01;1.41)	Reference	2.65 (2.20;3.51)	1.20 (1.02;1.41)	Reference	1.29 (0.84;1.97)	0.80 (0.65;0.97)
Heart Failure	1.60 (1.27;2.02)	1.04 (0.93;1.16)	Reference	1.27 (0.90;1.81)	1.19 (1.07;1.33)	Reference	1.26 (0.83;1.92)	0.88 (0.75;1.02)
Arrythmias	1.09 (0.96;1.24)	1.05 (1.00;1.11)	Reference	0.87 (0.71;1.07)	0.99 (0.93;1.05)	Reference	1.25 (0.98;1.60)	1.06 (0.99;1.15)
Intermittent claudication	2.33 (1.80;3.00)	1.46 (1.27;1.67)	Reference	2.96 (2.14;4.07)	1.77 (1.57;2.01)	Reference	0.79 (0.53;1.18)	0.82 (0.68;0.99)
Hypertension	1.60 (1.47;1.74)	1.27 (1.23;1.32)	Reference	1.03 (0.91;1.17)	1.29 (1.25,1.34)	Reference	1.55 (1.33;1.81)	0.98 (0.94;1.04)
Hyperlipidemia	1.39 (1.27;1.52)	1.16 (1.12;1.21)	Reference	1.12 (0.99;1.28)	1.10 (1.06;1.14)	Reference	1.24 (1.06;1.44)	1.05 (1.00;1.11)
**BMI categories**								
Normal weight	Reference	Reference	Reference	Reference	Reference	Reference	Reference	Reference
Underweight	1.69 (1.19;2.40)	0.75 (0.65;0.86)	Reference	2.76 (1.13;6.71)	2.17 (1.61;2.93)	Reference	0.61 (0.24;1.57)	0.34 (0.25;0.48)
Overweight	2.01 (1.82;2.23)	1.40 (1.35;1.45)	Reference	1.26 (1.10;1.44)	1.28 (1.23;1.33)	Reference	1.60 (1.35;1.90)	1.09 (1.04;1.15)
Obesity	3.96 (3.58;4.38)	1.79 (1.72;1.87)	Reference	2.19 (1.89;2.54)	1.85 (1.76;1.93)	Reference	1.81 (1.51;2.17)	0.97 (0.91;1.03)
**Blood pressure, ≥140/90 mm Hg**	1.32 (1.21;1.45)	1.20 (1.16;1.25)	Reference	1.08 (0.97;1.22)	1.21 (1.17;1.26)	Reference	1.22 (1.05;1.41)	0.99 (0.94;1.04)
**Smoking status**								
Never	Reference	Reference	Reference	Reference	Reference	Reference	Reference	Reference
Former	1.01 (0.91;1.13)	1.25 (1.20;1.29)	Reference	1.49 (1.29;1.74)	1.58 (1.52;1.64)	Reference	0.68 (0.57;0.81)	0.79 (0.75;0.83)
Current	2.93 (2.65;3.24)	1.79 (1.71;1.87)	Reference	4.36 (3.74;5.08)	2.47 (2.37;2.58)	Reference	0.67 (0.56;0.81)	0.72 (0.68;0.77)
**Alcohol consumption**								
Never	Reference	Reference	Reference	Reference	Reference	Reference	Reference	Reference
Former	0.18 (0.15;0.22)	0.89 (0.80;1.00)	Reference	0.20 (0.16;0.25)	1.20 (1.05;1.37)	Reference	0.90 (0.67;1.20)	0.74 (0.62;0.88)
Current (AUDIT-C score >3 in women, >4 in men)	0.04 (0.04;0.05)	0.56 (0.52;0.61)	Reference	0.03 (0.03;0.04)	0.64 (0.58;0.65)	Reference	1.41 (1.15;1.73)	0.88 (0.77;1.00)
Current (AUDIT-C score ≤3 in women, ≤4 in men	0.06 (0.05;0.07)	0.64 (0.60;0.69)	Reference	0.03 (0.03;0.04)	0.59 (0.53;0.65)	Reference	1.81 (1.51;2.17)	1.09 (0.96;1.24)
**Medication intake**								
Antihypertensive therapy	1.68 (1.54;1.84)	1.34 (1.29;1.39)	Reference	1.11 (0.97;1.28)	1.36 (1.30;1.41)	Reference	1.51 (1.28;1.78)	0.99 (0.94;1.04)
Beta-blockers	1.53 (1.37;1.72)	1.31 (1.25;1.38)	Reference	1.29 (1.07;1.55)	1.43 (1.36;1.51)	Reference	1.19 (0.96;1.48)	0.92 (0.85;0.99)
**Family history of MI**								
<60 years	1.71 (1.42;2.06)	1.17 (1.11;1.24)	Reference	1.76 (1.43;2.17)	1.16 (1.09;1.24)	Reference	0.97 (0.78;1.22)	1.01 (0.93;1.09)

*Analysis based on the population aged 40-69 years. BMI, body mass index; CVD, cardiovascular disease; MI, myocardial infarction

Table S9. Associations between education and several cardiovascular risk factors in the NAKO population 40-69 years old eligible for the SCORE2 algorithm NAKO study-baseline assessment. Presented are age-adjusted sex-specific ß-coefficients with 95% confidence intervals (CI) from linear regression models and women to men slope differences.

	Women N=73,903			Men N=69,116			Women to Men differences (95%CI)	
**Cardiovascular risk factor**	**Low education β (95% CI)**	**Middle education β (95% CI)**	**High education**	**Low education β (95% CI)**	**Middle education β (95% CI)**	**High education**	**Low education β_{women}- β_{men} (95% CI)**	**Middle education β_{women}- β_{men} (95% CI)**
**Blood pressure (BP), mmHg**								
Systolic BP	2.57 (1.92;3.22)	1.90 (1.65;2.14)	Reference	-0.50 (-1.35;0.34)	1.41 (1.17;1.65)	Reference	3.07 (1.99;4.14)	0.49 (0.15;0.84)
Diastolic BP	0.96 (0.57;1.36)	0.97 (0.82;1.11)	Reference	0.22 (-0.31;0.76)	0.95 (0.79;1.11)	Reference	0.74 (0.08;1.39)	0.01 (-0.20;0.23)
**Anthropometric measurements**								
Waist circumference (cm)	6.31 (6.79;6.82)	2.71 (2.51;2.90)	Reference	2.97 (2.30;3.64)	2.52 (2.33;2.70)	Reference	3.34 (2.45;4.22)	0.19 (-0.08;0.46)
Body mass index (kg/m^2)[2]	1.11 (1.10;1.12)	1.05 (1.04;1.05)	Reference	1.05 (1.04;1.05)	1.03 (1.03;1.04)	Reference	0.06 (0.06;0.05)	0.01 (0.01;0.02)
Body fat (%)	3.93 (3.65;4.21)	1.75 (1.64;1.86)	Reference	2.28 (1.91;2.64)	1.19 (1.09,1.30)	Reference	1.65 (1.17;2.12)	0.55 (0.40;0.69)
**Biomarkers:**								
Total cholesterol (mmol/l)	0.001 (-0.04;0.04)	0.02 (0.01;0.04)	Reference	-0.06 (-0.12;-0.01)	0.06 (0.05;0.08)	Reference	0.06 (-0.01;0.13)	-0.04 (-0.06;-0.02)
LDL-cholesterol (mmol/l) [1]	0.08 (0.05;0.12)	0.04 (0.03;0.05)	Reference	0.005 (-0.05;0.06)	0.05 (0.04;0.07)	Reference	0.08 (0.02;0.14)	-0.01 (-0.03;0.005)
HDL-cholesterol (mmol/l)	-0.16 (-0.18;-0.15)	-0.45 (-0.04;-0.03)	Reference	-0.12 (-0.14;-0.10)	-0.03 (-0.03;-0.02)	Reference	-0.04 (-0.07;-0.01)	-0.01 (-0.02;0.002)
HbA1c (mmol/mol) [2]	1.08 (1.07;1.08)	1.02 (1.02;1.02)	Reference	1.05 (1.05;1.06)	1.02 (1.01;1.02)	Reference	-0.01 (-0.02;-0.001)	-0.003 (-0.01;0.001)
Triglycerides (mmol/l)[2,3]	1.13 (1.11;1.16)	1.05 (1.04;1.06)	Reference	1.10 (1.07;1.14)	1.06 (1.05;1.07)	Reference	0.03 (-0.009;0.06)	-0.01 (-0.02;0.0003)
hs-CRP (mg/l)[2,4]	1.63 (1.55;1.73)	1.24 (1.21;1.26)	Reference	1.57 (1.46;1.68)	1.23 (1.20;1.25)	Reference	0.04 (-0.05;0.13)	0.01 (-0.02;0.04)

Beta estimates represent arithmetic differences in low and medium educational levels vs. high except for body mass index, HbA1c, triglycerides and hs-CRP in which estimates are interpreted as the ratio of the geometric mean of the outcome in low or middle education over the geometric mean of the outcome in high education.

[1] Data from 129,097 study participants

[2] Analyzed at the log scale; estimates were back transformed

[3] Data from 121,100 study participants

[4] Data from 85,370 study participants

Table S10. Sensitivity analyses - Associations between educational attainment and relative income with a very high-10-year risk of CVD in women and men 40-69 years old eligible for the SCORE2 algorithm (n=143,019, 51.7% women, 48.3% men). Presented are odds ratios (OR) and women to men ratio of OR (ROR) with 95% CI for low and middle education (reference=high education) and for low and middle relative income categories (reference=high relative income) from logistic models according to indicators of SEP.

	Women OR (95% CI)	Men OR (95% CI)	Women to Men ROR (95%CI)
**With migration background**			
n[a]	12,040	11,293	
**Educational attainment**			
Low	2.59 (1.62;4.14)	1.28 (1.02;1.61)	2.02 (1.20;3.41)
Medium	1.82 (1.29;2.55)	1.29 (1.15;1.45)	1.41 (0.98;2.02)
High	Reference	Reference	Reference
**Relative income**			
<60 %	2.78 (1.56;4.95)	1.99 (1.69;2.34)	1.40 (0.77;2.55)
60 % -79 %	2.96 (1.65;5.33)	1.53 (1.26;1.87)	1.93 (1.04;3.59)
80 % -99 %	1.69 (0.89;3.19)	1.22 (1.00;1.50)	1.39 (0.71;2.71)
100 % -149 %	1.48 (0.82;2.66)	1.15 (0.97;1.37)	1.29 (0.70;2.38)
≥150 %	Reference	Reference	Reference
**Without migration background**			
n[a]	61,863	57,824	
**Educational attainment**			
Low	4.53 (3.49;5.89)	2.25 (1.86;2.72)	2.01 (1.46;2.78)
Medium	1.71 (1.48;1.96)	1.28 (1.22;1.34)	1.34 (1.15;1.55)
High	Reference	Reference	Reference
**Relative income**			
<60 %	3.02 (2.42;3.78)	2.40 (2.22;2.60)	1.26 (0.99;1.59)
60 % -79 %	2.85 (2.31;3.52)	2.02 (1.87;2.17)	1.41 (1.13;1.76)
80 % -99 %	2.10 (1.68;2.62)	1.37 (1.27;1.48)	1.53 (1.21;1.94)
100 % -149 %	1.50 (1.22;1.84)	1.25 (1.18;1.33)	1.20 (0.96;1.50)
≥150 %	Reference	Reference	Reference
**Employed**			
n[a]	55,797	55,727	
**Educational attainment**			
Low	3.19 (1.95;5.21)	1.51 (1.22;1.88)	2.11 (1.20;3.71)
Medium	1.33 (1.05;1.69)	1.36 (1.28;1.44)	0.98 (0.77;1.25)
High	Reference	Reference	Reference
**Relative income**			
<60 %	1.87 (1.27;2.76)	1.66 (1.79;1.85)	1.13 (0.76;1.66)
60 % -79 %	1.69 (1.19;2.41)	1.33 (1.21;1.47)	1.27 (1.11;1.46)
80 % -99 %	1.20 (0.83;1.74)	1.18 (1.08;1.29)	1.02 (0.70;1.49)
100 % -149 %	1.13 (0.83;1.54)	1.04 (0.97;1.11)	1.09 (0.79;1.49)
≥150 %	Reference	Reference	Reference
**Unemployed[b]**			
n[a]	1,895	2,435	
**Educational attainment**			
Low	1.72 (0.55;5.38)	1.43 (0.97;2.10)	1.20 (0.36;4.01)
Medium	1.20 (0.51;2.82)	1.25 (0.98;1.59)	0.96 (0.39;2.33)
High	Reference	Reference	Reference
**Economically inactive**			
n[a]	16,210	10,955	
**Educational attainment**			
Low	1.87 (1.43;2.44)	0.95 (0.73;1.23)	1.97 (1.36;2.86)
Medium	1.41 (1.20;1.65)	0.99 (0.92;1.08)	1.42 (1.19;1.70)
High	Reference	Reference	Reference
**Relative income**			
<60 %	1.59 (1.21;2.08)	1.04 (0.92;1.18)	1.53 (1.13;2.06)
60 % -79 %	1.81 (1.40;2.34)	1.35 (1.19;1.53)	1.34 (1.01;1.78)
80 % -99 %	1.89 (1.44;2.49)	1.21 (1.05;1.39)	1.56 (1.15;2.12)

100 % -149 %	1.47 (1.14;1.91)	1.20 (1.07;1.35)	1.23 (0.92;1.63)
≥150 %	Reference	Reference	Reference

[a] Numbers correspond to the average estimates across all the imputations.
[b]Few numbers of persons in some strata of relative income precluded the analysis in the unemployed group.

Table S11. Median (IQR) for numerical cardiovascular risk factors and percentages for binary risk factors in participants of the NAKO study baseline assessment eligible for the PCE algorithm*

Risk factor	All n=140,464	Women n=71,355	Men n=69,109
Age (years)	54 (47-62)	54 (48-62)	53 (47-62)
Current smokers	19.29	17.95	20.67
SBP (mmHg)	128 (118-140)	124 (114-136)	132 (123-142)
Total cholesterol (mmol/l)	5.45 (4.80-6.17)	5.50 (4.89-6.23)	5.40 (4.71-6.10)
HDL-cholesterol (mmol/l)	1.50 (1.23-1.81)	1.70 (1.44-2.01)	1.32 (1.11-1.56)
Antihypertensive treatment	27.07	25.15	29.05
Diabetes mellitus[a]	7.12	6.12	8.16
Diabetes mellitus[b]	6.18	5.49	6.89
**Risk factor**	**Caucasians[c] n=126,817**	**Women n=63,907**	**Men n=62,910**
Age (years)	53 (47-62)	54 (47-62)	53 (47-62)
Current smokers	18.65	17.37	19.94
SBP (mmHg)	128 (118-139)	124 (114-136)	132 (123-142)
Total cholesterol (mmol/l)	5.43 (4.80-6.15)	5.50 (4.87-6.20)	5.40 (4.71-6.10)
HDL-cholesterol (mmol/l)	1.50 (1.24-1.82)	1.71 (1.45-2.01)	1.32 (1.11-1.56)
Antihypertensive treatment	26.43	24.46	28.42
Diabetes mellitus[a]	6.75	5.83	7.69
Diabetes mellitus[b]	5.86	5.24	6.48
**Risk factor**	**Africans[c] n=468**	**Women n=175**	**Men n=293**
Age (years)	48 (44-56)	48 (43-54)	49 (45-56)
Smoking status	16.0	14.86	16.72
SBP (mmHg)	128 (118-139)	123 (115-137)	130 (120-140)
Total cholesterol (mmol/l)	5.10 (4.45-5.72)	5.30 (4.60-5.88)	5.00 (4.30-5.60)
HDL-cholesterol (mmol/l)	1.46 (1.20-1.71)	1.64 (1.42-1.87)	1.34 (1.11-1.60)
Antihypertensive treatment	23.57	22.16	24.41
Diabetes mellitus[a]	11.54	10.29	12.29
Diabetes mellitus[b]	9.83	8.00	10.92

*Analysis based on the population aged 40-79 years.

[a]Diabetes mellitus defined as self-reported data or HbA1c ≥ 48 mmol/mol.

[b]Diabetes mellitus defined as self-reported data.

[c]Sex-specific frequencies of ethnicity/country of origin for the 204,780 study participants: Caucasians (98.68% women, 98.64% men), Africans (0.41% women, 0.63% men), South-east Asians (0.39% women, 0.21% men), Central-South Americans (0.21% women, 0.15% men), Others (0.31% women, 0.33% men).

Table S12. Distribution of socioeconomic variables in the eligible population for the PCE and Reynolds Score algorithms*

	Women	Men
**PCE (n=126,817) ***	63,907	62,910
**Educational attainment, %**		
Low	2.83	1.40
Medium	46.93	35.89
High	50.24	62.71
**Relative income, %**		
<60 %	11.46	9.14
60 % -79 %	15.53	11.65
80 % -99 %	15.67	13.19
100 % -149 %	32.58	33.04
≥150 %	24.75	32.98
**Employment status, %**		
Employed	75.11	79.29
Unemployed	2.13	2.86
Economically inactive	22.76	17.85
**Immigration background, %**	14.45	14.10
**Reynolds Score (n=60,073)**	30,237	29,836
**Educational attainment, %**		
Low	2.26	0.97
Medium	46.58	33.63
High	51.16	65.40
**Relative income, %**		
<60 %	10.04	7.97
60 % -79 %	14.63	10.68
80 % -99 %	14.79	12.41
100 % -149 %	33.29	32.90
≥150 %	27.27	36.04
**Employment status, %**		
Employed	75.72	79.91
Unemployed	1.99	2.63
Economically inactive	22.29	17.46
**Immigration background, %**	12.06	12.22

*Estimates only shown for those reported as Caucasians. PCE: Pooled Cohort Equation.
Number of missing data in the population eligible for the PCE: education (n=9,063), income (n=7,733), employment (772), migration (n=24).
Number of missing data in the population eligible for the Reynold Score algorithm: education (n=4,197), income (n=3,430), employment (403), migration (n=7).
*PCE: Analysis based on the population aged 40-79 years, Reynolds Score based on the population over 45 years old.

Table S13. **PCE risk score: Predicted 10-year risk of CVD (%)**

	Low risk<br><5%	Borderline risk<br>5 to <7.5%	Intermediate risk<br>≥7.5% to <20%	High-risk<br>≥20%
		**All (n=140,370) ***		
Women, n (%)	50,824 (71.26)	7,988 (11.20)	11,350 (15.91)	1,156 (1.62)
Men, n (%)	25,258 (36.58)	9,548 (13.83)	25,447 (36.88)	8,799 (12.74)
		**Caucasians (n=126,817)**		
Women, n (%)	46,137 (72.19)	7058 (11.04)	9789 (15.32)	923 (1.44)
Men, n (%)	23,605 (37.52)	8,794 (13.98)	22,960 (36.50)	7,551 (12.00)
		**Africans (n=468)**		
Women, n (%)	131 (74.68)	13 (7.43)	29 (16.57)	2 (1.14)
Men, n (%)	106 (36.18)	72 (24.57)	83 (28.33)	32 (10.92)

*Algorithm for Caucasian was applied. PCE, Pooled Cohort Equation; CVD, cardiovascular disease. *Analysis based on the population aged 40-79 years.

Table S14. Median (IQR) for numerical cardiovascular risk factors and percentages for binary risk factors in participants of the NAKO study baseline assessment eligible for the Reynolds Risk Score algorithm*

**Risk factor**	**All n=60,073**	**Women n=30,237**	**Men n=29,836**
Age (years)	55 (49-62)	55 (49-62)	54 (49-62)
Smoking status	17.39	16.42	18.38
SBP (mmHg)	129 (119-140)	125 (114-137)	132 (123-143)
Total cholesterol (mmol/l)	5.50 (4.90-6.20)	5.60 (5.00-6.30)	5.40 (4.80-6.10)
HDL-cholesterol (mmol/l)	1.54 (1.27-1.86)	1.75 (1.49-2.06)	1.35 (1.14-1.59)
hs-CRP (mg/l)	1.04 (0.56-2.23)	1.06 (0.55-2.39)	1.01 (0.56-2.09)
Family history of MI	9.71	10.25	9.16

* Reynolds Score algorithm based on the population over 45 years. IQR, interquartile range; SBP, systolic blood pressure; HDL-cholesterol, high-density lipoprotein cholesterol; hs-CRP, high sensitive C-reactive protein; MI, myocardial infarction

Table S15. Reynolds risk score*: Predicted 10-year risk of CVD (%)

	Low risk<br><5%	Borderline risk<br>5 to < 10%	Intermediate risk<br>≥10% to < 20%	High risk<br>≥20%
		All (n=60,073)		
Women, n (%)	26,867 (88.85)	2,633 (8.71)	656 (2.17)	81 (0.27)
Men, n (%)	12,524 (41.98)	9,329 (31.27)	6,412 (21.49)	1,571 (5.27)

* Reynolds Score algorithm based on the population over 45 years. CVD, cardiovascular disease

Table S16. Sex-specific odds ratios (OR) and women to men ratio of ORs with 95% CI for high predicted 10-year risk of CVD in relation to indicators of SEP using the Pooled cohort equation (PCE) and Reynolds risk score in the NAKO-baseline assessment.

	**Women OR (95% CI)**		**Men OR (95% CI)**		**Women to Men ROR (95%CI)**	
	**Primary model**	**Age-adjusted**	**Primary model**	**Age-adjusted**	**Primary model**	**Age-adjusted**
**EDUCATIONAL ATTAINMENT**						
**PCE** ≥10% *						
	n=59,226	n=59,226	n=58,528	n=58,528		
Low	3.22 (2.85;3.65)	2.16 (1.84;2.53)	1.43 (1.24;1.64)	3.14 (2.55;3.86)	2.25 (1.87;2.71)	0.69 (0.53;0.89)
Medium	1.57 (1.49;1.66)	1.45 (1.35;1.55)	1.14 (1.09;1.17)	1.65 (1.57;1.74)	1.38 (1.29;1.47)	0.88 (0.81;0.96)
High	Reference	Reference	Reference	Reference	Reference	Reference
**Reynolds Score** ≥10%						
	n=28,015	n=28,015	n=27,861	n=27,861		
Low	2.79 (1.93;4.04)	1.85 (1.25;2.73)	1.09 (0.83;1.42)	2.00 (1.41;2.84)	2.56 (1.62;4.04)	0.93 (0.55;1.56)
Medium	1.48 (1.26;1.73)	1.36 (1.16;1.61)	0.98 (0.92;1.03)	1.37 (1.27;1.48)	1.51 (1.28;1.79)	0.99 (0.83;1.19)
High	Reference	Reference	Reference	Reference	Reference	Reference
**RELATIVE INCOME**						
**PCE** ≥10% *						
	n=59,189	n=59,189	n=59,895	n=59,895		
<60 %	2.81 (2.55;3.09)	2.42 (2.15;2.71)	1.94 (1.83;2.07)	2.41 (2.19;2.64)	1.45 (1.29;1.62)	1.00 (0.87;1.17)
60 % -79 %	2.99 (2.74;3.27)	1.94 (1.74;2.15)	1.78 (1.68;1.88)	1.87 (1.71;2.04)	1.68 (1.51;1.87)	1.04 (0.90;1.19)
80 % -99 %	1.89 (1.72;2.08)	1.63 (1.46;1.83)	1.08 (1.02;1.14)	1.58 (1.46;1.72)	1.75 (1.57;1.95)	1.03 (0.90;1.19)
100 % -149 %	1.45 (1.33;1.58)	1.45 (1.31;1.60)	1.10 (1.06;1.15)	1.32 (1.24;1.40)	1.32 (1.20;1.45)	1.10 (0.98;1.23)
≥150 %	Reference	Reference	Reference	Reference	Reference	Reference
**Reynolds Score** ≥10%						
	n=28,120	n=28,120	n=28,523	n=28,523		
<60 %	2.11 (1.62;2.75)	1.77 (1.34;2.33)	1.53 (1.38;1.69)	1.66 (1.45;1.89)	1.38 (1.04;1.83)	1.07 (0.78;1.45)
60 % -79 %	1.92 (1.50;2.46)	1.41 (1.09;1.82)	1.44 (1.32;1.58)	1.35 (1.20;1.53)	1.33 (1.02;1.73)	1.04 (0.79;1.39)
80 % -99 %	1.75 (1.36;2.25)	1.48 (1.15;1.93)	1.01 (0.93;1.11)	1.35 (1.19;1.52)	1.73 (1.33;2.26)	1.10 (0.82;1.46)
100 % -149 %	1.34 (1.08;1.67)	1.29 (1.03;1.61)	1.09 (1.02;1.16)	1.23(1.13;1.33)	1.23 (0.98;1.54)	1.05 (0.83;1.33)
≥150 %	Reference	Reference	Reference	Reference	Reference	Reference

*In the population reported as Caucasians. CVD, Cardiovascular Disease; PCE, Pooled Cohort Equation.
PCE based on the population aged 40-79 years, Reynolds Score algorithm based on the population over 45 years old.

Figure S1: Flowchart illustrating the inclusion criteria for the SCORE2 algorithm risk score calculation

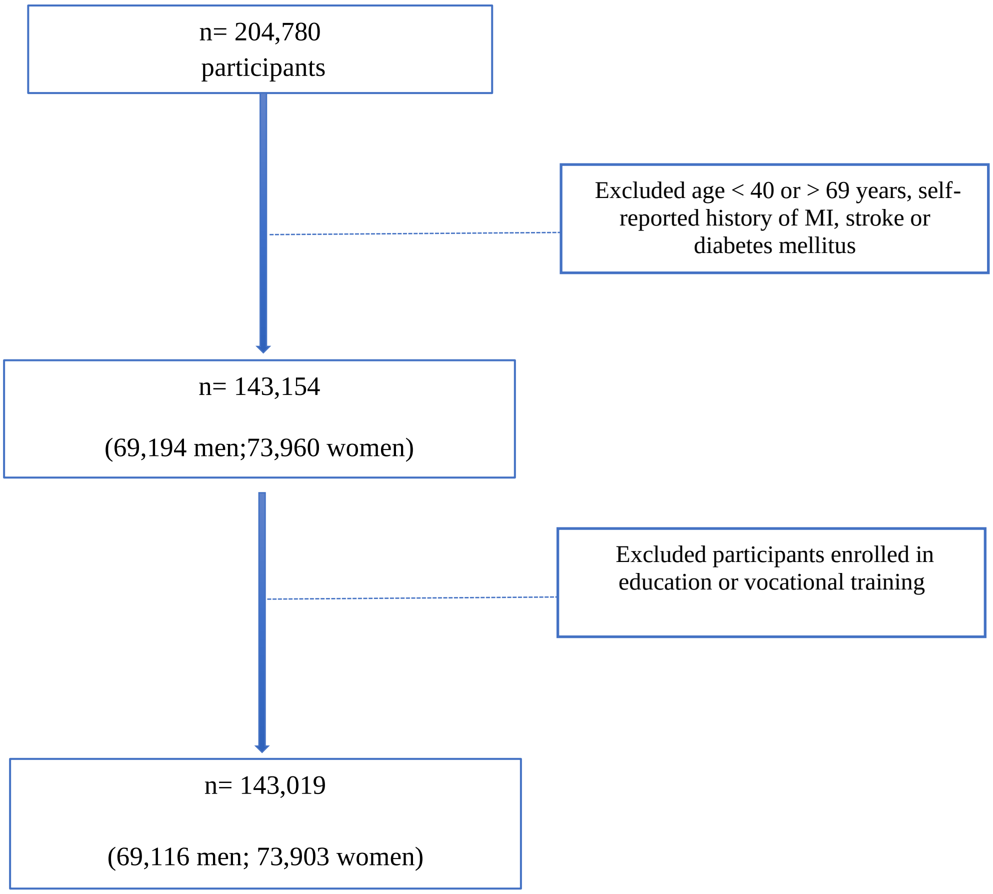

MI, myocardial infarction

Figure S2. Flowchart illustrating the inclusion criteria for the Pooled cohort equation (PCE) algorithm risk score calculation*

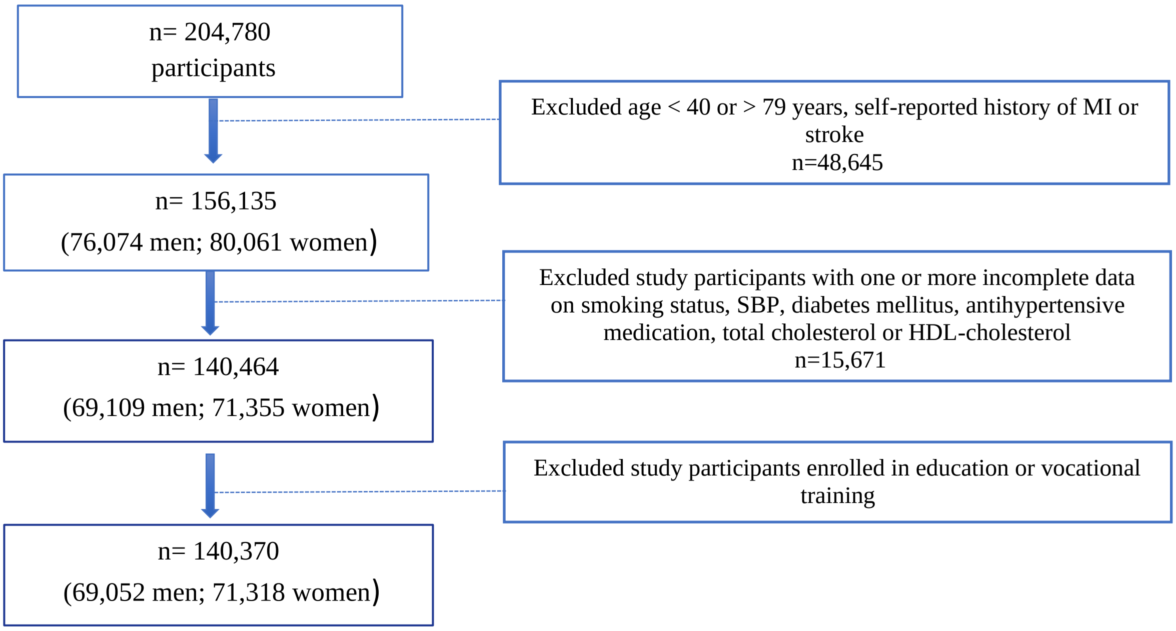

MI, myocardial infarction; SBP, systolic blood pressure.

*The PCE estimates the 10-year risk of atherosclerotic CVD (non-fatal MI or coronary heart disease (CHD) death, fatal or nonfatal stroke) in the CVD free population in both sexes. The PCE which can be used in individuals 40 to 79 years old, incorporates ethnicity (non-Hispanic African Americans and non-Hispanic whites) in addition to the traditional cardiovascular risk factors: sex, age, SBP (mmHg), antihypertensive treatment (yes/no), total cholesterol (mg/dl), HDL-cholesterol (mg/dl), diabetes mellitus (yes/no) and current smoking (yes/no). Values using the international system of units (SI) mmol/l were converted to the unit system required in the algorithm (mg/dl).

Figure S3. Flowchart illustrating the inclusion criteria for the Reynolds Risk Score algorithm*

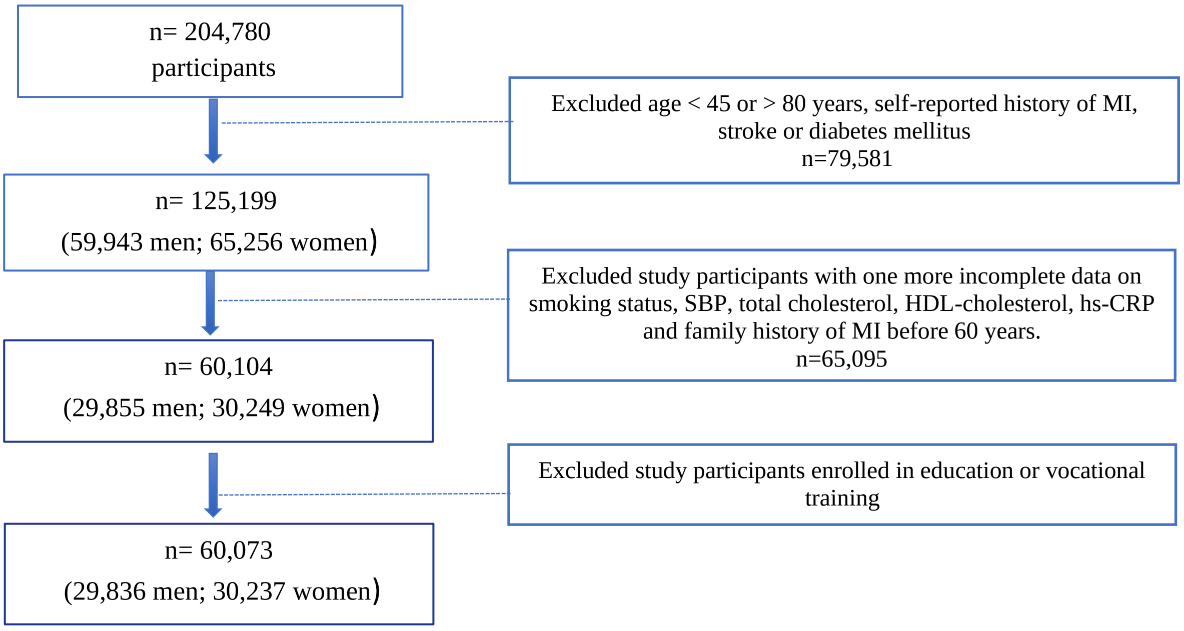

MI, myocardial infarction; SBP, systolic blood pressure; hs-CRP, high sensitive C-reactive protein.

The Reynolds Risk Score estimates the 10-year CVD risk (MI, ischemic stroke, coronary revascularization and cardiovascular death) in women and men. Factors in the risk prediction model included age (years), smoking (currents vs. other), SBP (mmHg), total cholesterol (mg/dl), HDL-cholesterol (mg/dl), hs-CRP (mg/l) and family history (parental) of premature MI (yes/no). Values using the international system of units (SI) mmol/l were converted to the unit system required in the algorithm (mg/dl).

Figure S4. Associations between relative income and CVD or CVD risk factors in the NAKO study-baseline assessment in women and men (n=200,279, 50.5% women, 49.5% men)*. Presented are age-adjusted odds ratios (OR) with 95% CI for relative income <60%, 60%-79%, 80%-99% and 100%-149% (reference=high relative income) from logistic or multinomial regression models

(A)

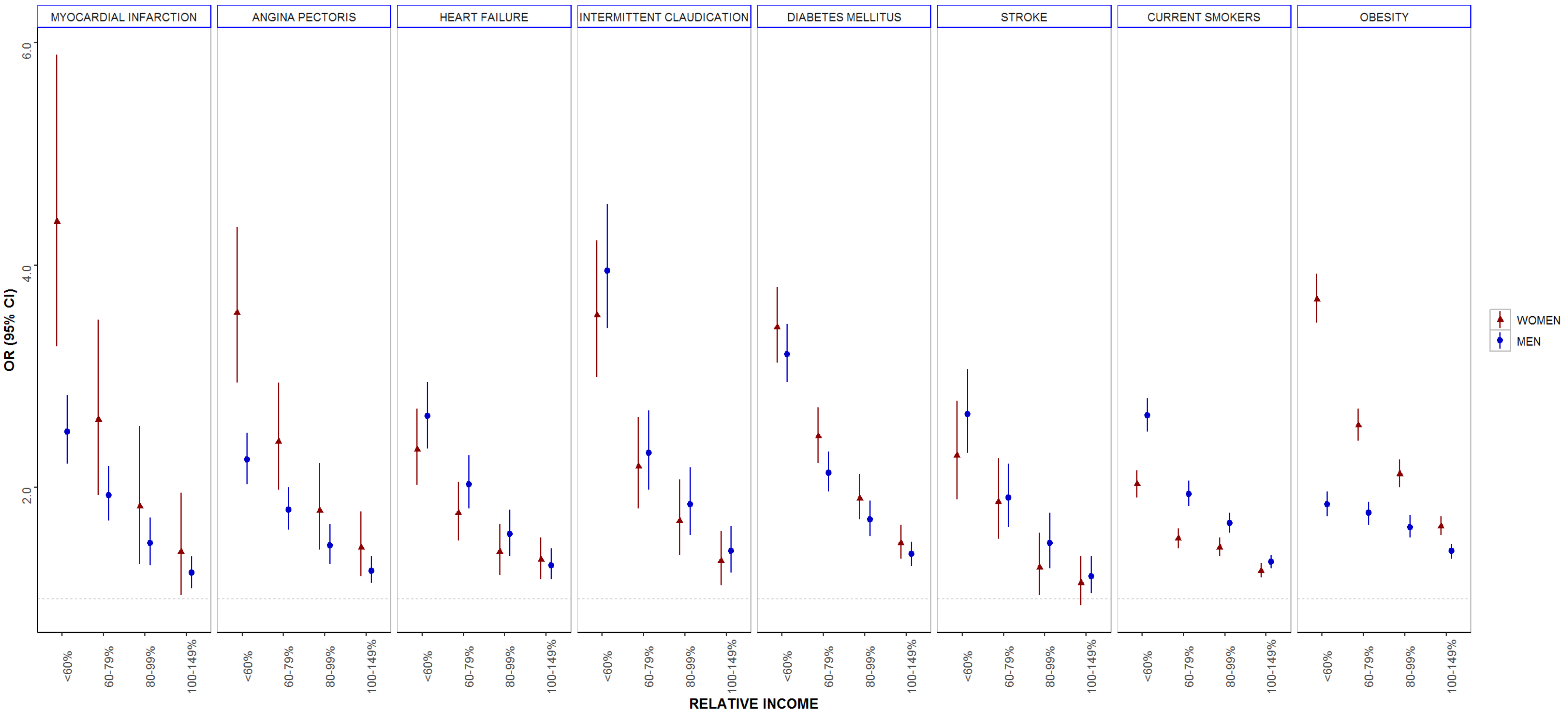

(B)

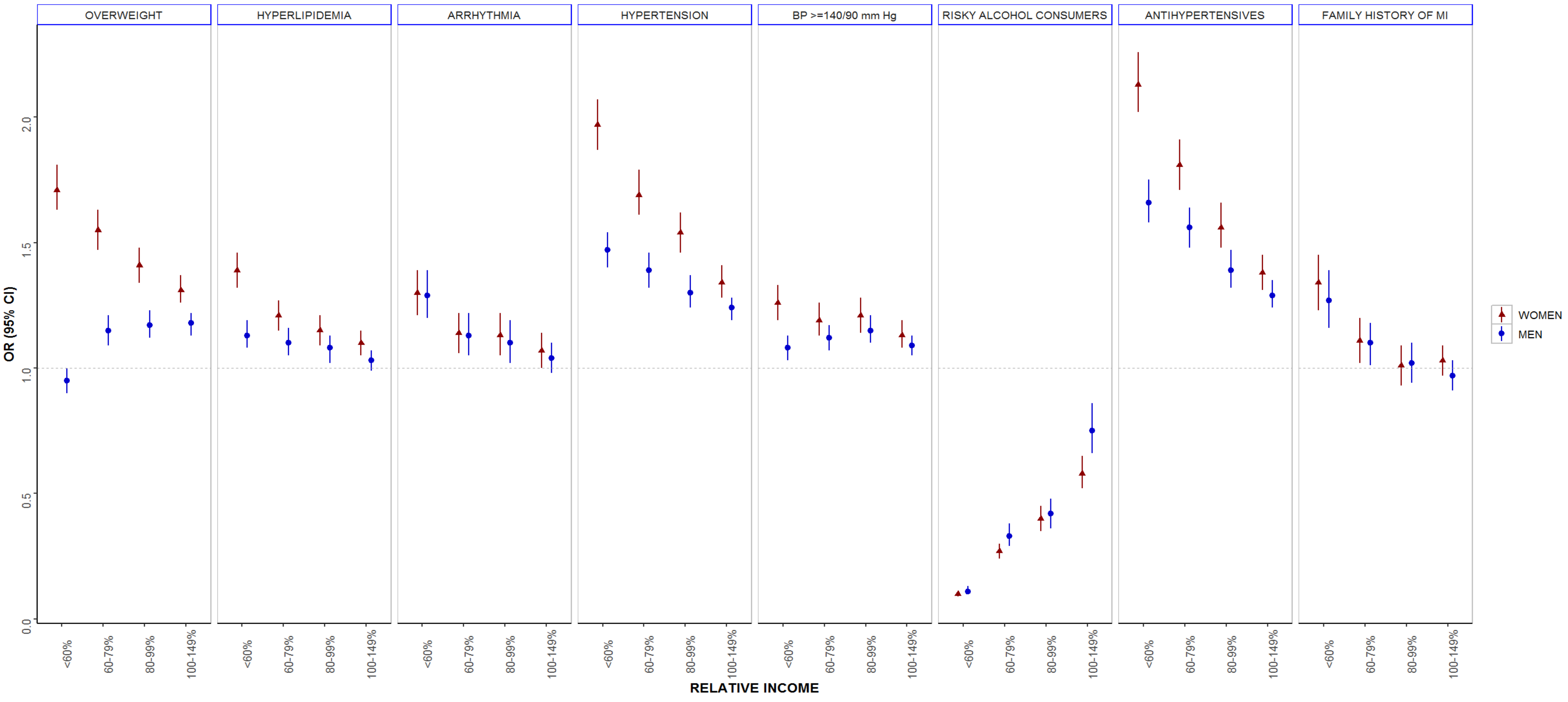

*Analysis based on the entire population at baseline. BP, blood pressure; MI, myocardial infarction
Reference categories in the multinomial logistic regression models for BMI categories (normal weight), smoking status (never) and alcohol consumption (never).

Figure S5. Frequencies of predicted 10-year CVD risk in women and men 40-69 years old from the NAKO study-baseline assessment using the SCORE2 algorithm

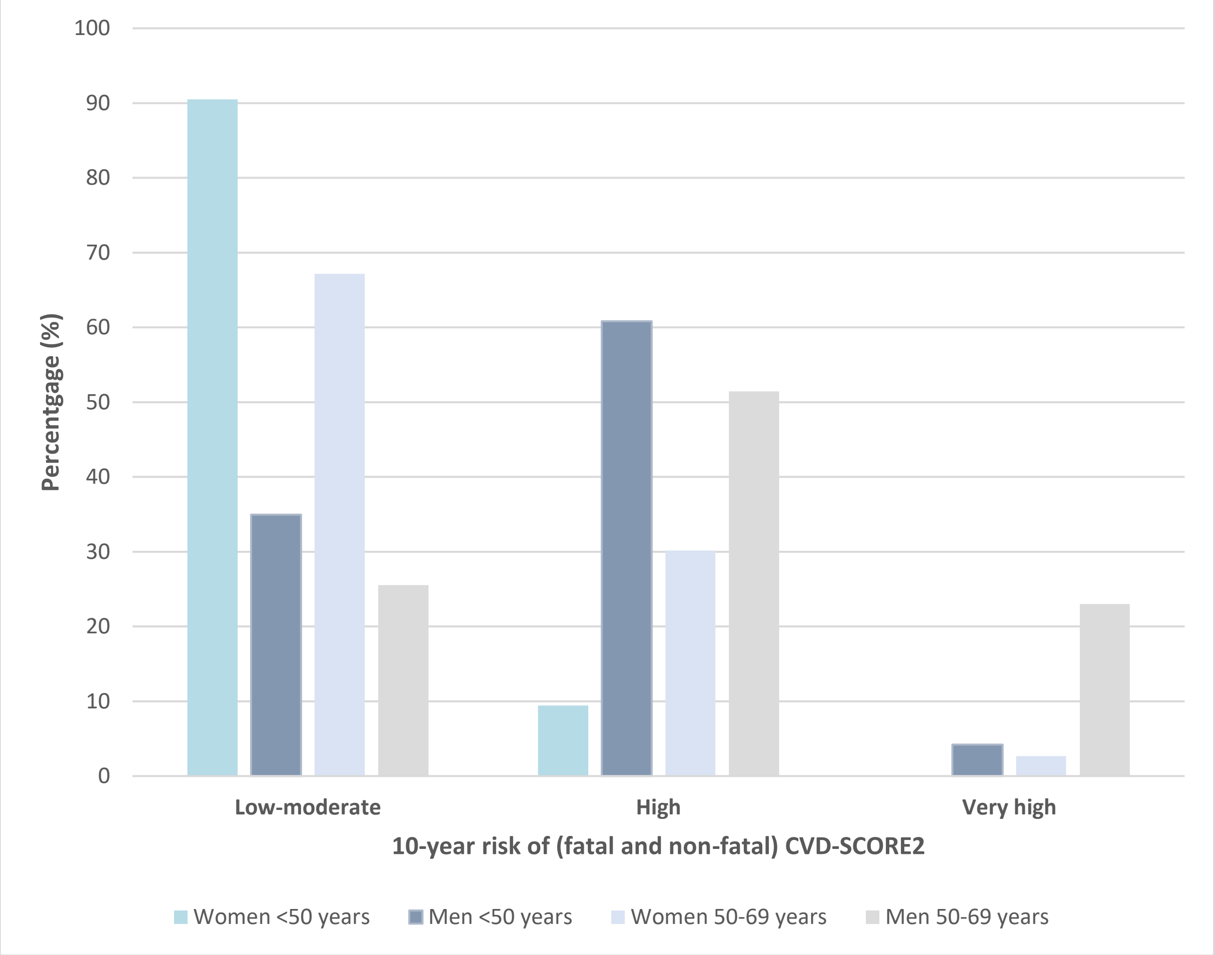

<50 years	Low-moderate risk (<2.5 %)	High-risk (2.5 to <7.5%)	Very high-risk (≥7.5%)
Number of women, n*	22,912	2,392	17
Number of men, n*	8,852	15,375	1,068
**50-69 years**	**Low-moderate risk (<5 %)**	**High-risk 5 to (<10%)**	**Very high-risk (≥10%)**
Number of women, n*	32,618	14,661	1,302
Number of men, n*	11,191	22,552	10,079

* Numbers correspond to the average estimates across all the imputations.

Figure S6. Odds ratio (95% confidence intervals) for the association between proxies of SES (A) educational attainment, (B) relative income) and very high-risk SCORE2 for predicted 10-year risk of CVD in the NAKO study* (reference category highly educated women).

A.

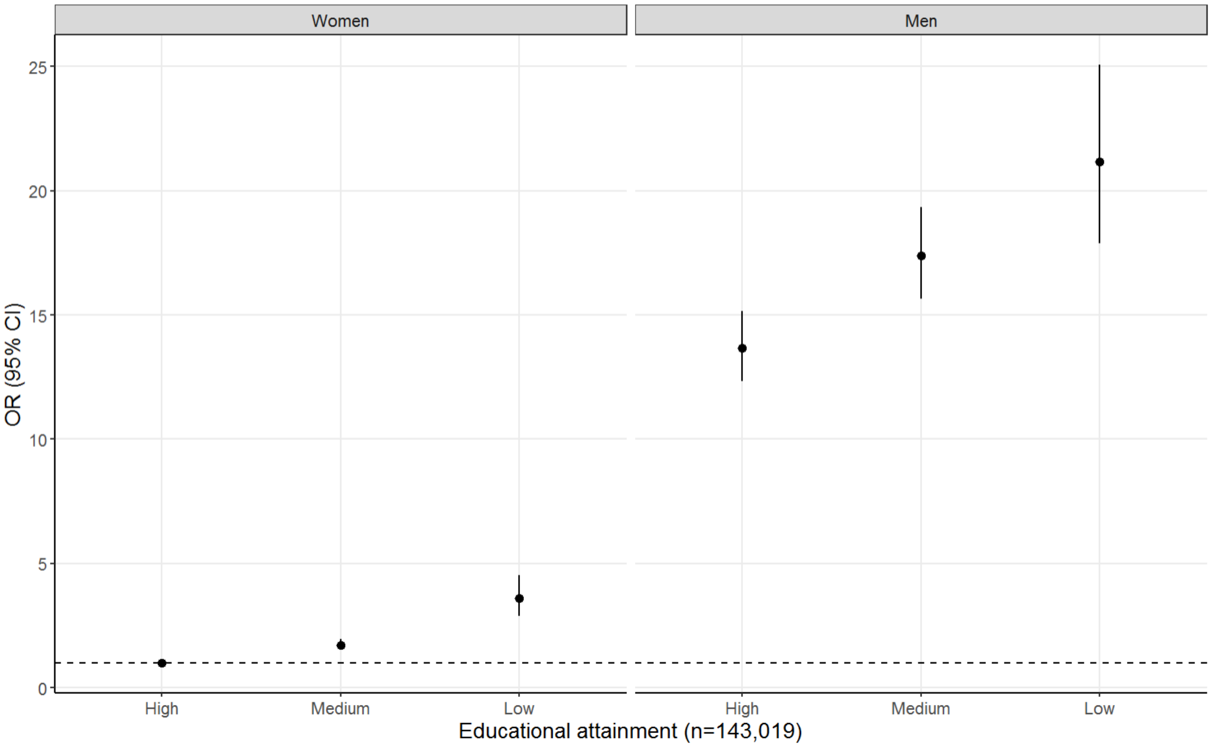

B.

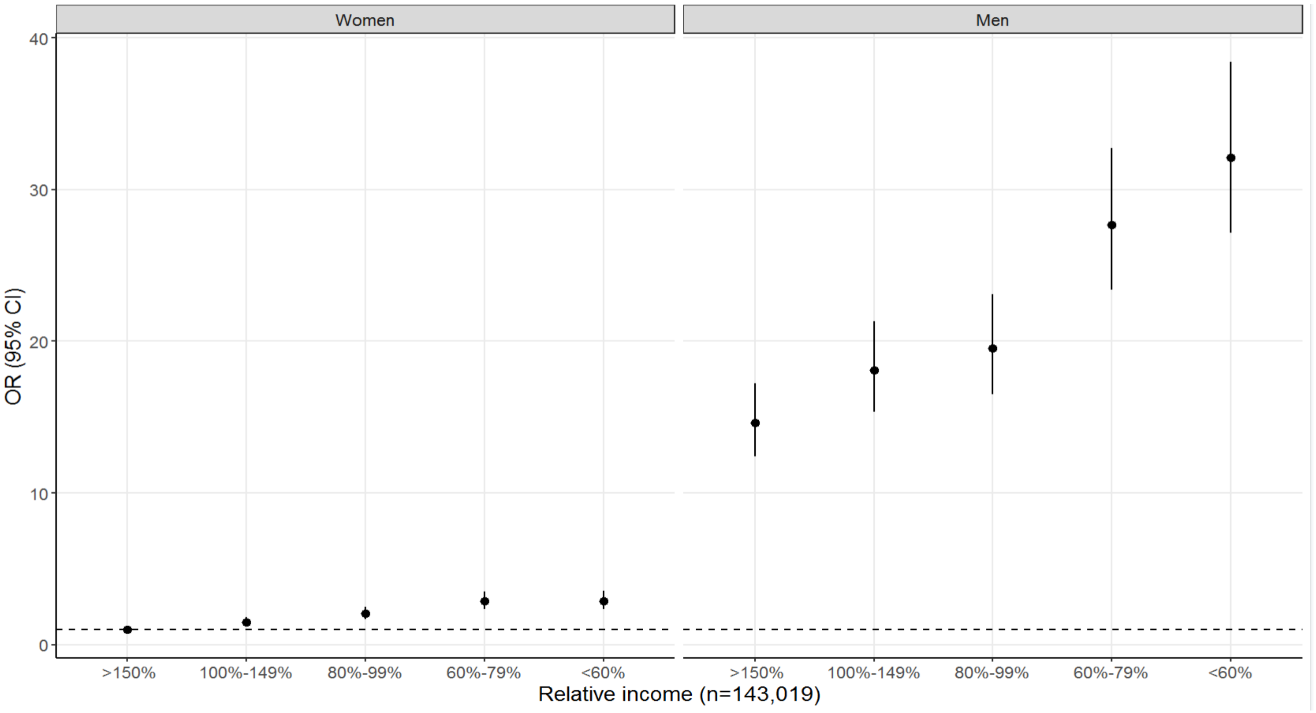

* Analysis based on the population 40-69 years.
